# A Bioinformatics-Based Alternative mRNA Splicing Code that May Explain Some Disease Mutations Is Conserved in Animals

**DOI:** 10.3389/fgene.2017.00038

**Published:** 2017-04-11

**Authors:** Wen Qu, Pablo Cingolani, Barry R. Zeeberg, Douglas M. Ruden

**Affiliations:** ^1^Department of Pharmacology, Wayne State UniversityDetroit, MI, USA; ^2^Department of Obstetrics and Gynecology, Wayne State UniversityDetroit, MI, USA; ^3^Genome Quebec Innovation Centre, School of Computer Science, McGill UniversityQC, Canada; ^4^Genomics and Bioinformatics Group, Laboratory of Molecular Pharmacology, Center for Cancer Research, National Cancer Institute, National Institutes of HealthBethesda, MD, USA; ^5^Institute of Environmental Health Sciences, Wayne State UniversityDetroit, MI, USA

**Keywords:** alternative splicing, RNA metabolism, bioinformatics, splicesosome

## Abstract

Deep sequencing of cDNAs made from spliced mRNAs indicates that most coding genes in many animals and plants have pre-mRNA transcripts that are alternatively spliced. In pre-mRNAs, in addition to invariant exons that are present in almost all mature mRNA products, there are at least 6 additional types of exons, such as exons from alternative promoters or with alternative polyA sites, mutually exclusive exons, skipped exons, or exons with alternative 5′ or 3′ splice sites. Our bioinformatics-based hypothesis is that, in analogy to the genetic code, there is an “alternative-splicing code” in introns and flanking exon sequences, analogous to the genetic code, that directs alternative splicing of many of the 36 types of introns. In humans, we identified 42 different consensus sequences that are each present in at least 100 human introns. 37 of the 42 top consensus sequences are significantly enriched or depleted in at least one of the 36 types of introns. We further supported our hypothesis by showing that 96 out of 96 analyzed human disease mutations that affect RNA splicing, and change alternative splicing from one class to another, can be partially explained by a mutation altering a consensus sequence from one type of intron to that of another type of intron. Some of the alternative splicing consensus sequences, and presumably their small-RNA or protein targets, are evolutionarily conserved from 50 plant to animal species. We also noticed the set of introns within a gene usually share the same splicing codes, thus arguing that one sub-type of splicesosome might process all (or most) of the introns in a given gene. Our work sheds new light on a possible mechanism for generating the tremendous diversity in protein structure by alternative splicing of pre-mRNAs.

## Introduction

The almost invariant consensus sequence for mRNA splicing in animals and plants is gu_ag, where gu is the splice donor sequence and ag is the splice acceptor sequence. A longer splice donor consensus sequence in most mammals is guragu, where r is either g or a (Mount, 1982; Black, 2003). Usually, an expression of “gu_ag” means that only the 5′ and 3′ terminal two nucleotides of the sequence are invariable as gu and ag, respectively, and that a sequence represented by the underscore can be any sequences. However, here we use this expression to indicate that the sequence represented by the underscore can be any sequences except for sequences that do not match any of the other consensus sequences.

The splice acceptor consensus sequence is preceded by a branch point sequence, which contains an adenine, which is ligated to the 5′ splice site ribonucleotide to form the intron lariat, and a polypyrimidine tract (c or u), which is between the branch point and the splice acceptor sequence. While the short gu_ag consensus sequence of introns is clearly not sufficient to differentiate amongst the multitude of alternative splicing events, surprisingly little is known about what other sequence information is required to regulate alternative RNA splicing (Ladd and Cooper, 2002; Barash et al., 2010; Witten and Ule, 2011). The flanking one or two nucleotides on either side of the intron are also often conserved, and they are included in our supplementary tables, but they will not be discussed further in this paper so that we can focus our analyses on consensus sequences at the ends of the introns.

Alternative RNA splicing occurs in almost all human genes and vastly increases the number of proteins and transcripts that an organism can produce (Pan et al., 2008; Wang et al., 2008). Exons that are involved in all types of RNA splicing can be classified into five major categories: (1) exons containing alternative 5′ splice sites (A5), (2) exons containing alternative 3′ splice sites (A3), (3) retained or invariant exons (R), (4) skipped exons (S), and (5) mutually exclusive exons (ME) (Ast, 2004; Sugnet et al., 2004). In addition to these five types of exons, we also include in our bioinformatics analysis exons that contain an alternative promoter (APr) and exons that contain an alternative poly A (APA) site to make a total of 7 exon types. Since an intron is flanked by two exons, APr can only be at the 5′ end, and APA can only be at the 3′ end, there are 36 possible pair-wise types of introns that are distinguished by the combinations of 7 types of flanking exons. Here we present bioinformatics evidence to support our hypothesis that there is an alternative splicing code based on the 36 different types of introns.

## Results

We hypothesize that there are 36 different types of introns with unique consensus sequences based on their flanking exon types. To test this hypothesis, we analyzed the 36 different types of introns separately rather than combining all introns into one pool, as is usually done in bioinformatics studies of introns (e.g., Mount, 1982). It is possible, for instance, that there are many different types of macromolecular splicesosome complexes, in addition to the canonical U2-type splicesosomes and the non-canonical U12-type splicesosomes (Padgett, 2012), that utilize the numerous U2-variant small RNA sequences in the genome (O'Reilly et al., 2013). To test this hypothesis, we determined whether the 36 types of introns are enriched for a particular paired consensus sequence(s) that is derived from both ends of the intron and flanking exon regions. Supplementary Table 1 has a list of all 36 types of introns and the number of introns in each class in *H. sapiens, M. musculis, D. melanogaster, C. elegans*, and *A. thaliana*.

To begin our bioinformatics analysis of introns, we first generated a table of paired splice donor and acceptor consensus sequences, from the most common to the least common (see Materials and Methods). For statistical reasons, we selected an arbitrary cutoff of each paired consensus sequence being represented by at least 100 introns. Using a modification of our program SnpEff, which classifies sequences in any sequenced genome (Cingolani et al., 2012), we analyzed the genomes and compared them with annotated full-length RefSeq cDNAs of 50 different plant and animal species. The total number of different types of paired consensus sequences ranged from one in baker's yeast, *Saccharomyces cerevisiae*, guaugu_ag, which has only 282 introns, all of which are always flanked by invariant exons (R-R), to 95 different consensus sequences in the marmoset, which has 184,882 introns (Supplementary Table 2). The average number of introns in the 50 species that we analyzed was 116,288 with a standard deviation of 45,266. Almost half of the animals' genomes that we analyzed have between 40 and 50 different types of paired consensus sequences with at least 100 introns in each type. Table 1 shows the 42 consensus sequences in humans, which are in at least 100 introns, in rank order from most common to least common.

**Table 1:** **The top 42 ranked intron consensus sequences in humans**.

**Rank**	**5S-3S**	**Count**
0	ALL	215,155
1	gugagu_ag	30,585
2	guaag_ag	29,538
3	guaagu_ag	28,972
4	gugag_ag	26,627
5	guaa_ag	22,188
6	gua_ag	20,040
7	guagg_ag	12,474
8	guaugu_ag	6,312
9	guaug_ag	5,552
10	guggg_cag	5,168
11	gu_ag	4,901
12	guga_ag	3,332
13	gucagu_ag	2,439
14	gugcg_cag	1,904
15	gucag_ag	1,650
16	gugugu_ag	1,421
17	guuagu_ag	1,415
18	guuugu_ag	1,113
19	gcaagu_ag	967
20	guuggu_ag	929
21	gugggu_ag	918
22	gugug_ag	912
23	guggg_ag	719
24	gucugu_ag	450
25	gucug_ag	371
26	gugcgu_ag	311
27	gcaag_ag	301
28	gugcg_ag	255
29	guauccuuu_ag	250
30	gc_ag	230
31	gucgg_cag	202
32	guca_ag	198
33	guccg_ag	162
34	gcagg_ag	153
35	guucgu_ag	147
36	guauccuu_ag	144
37	auauccuu_ac	124
38	gua_ugguuucag	118
39	guaag_uguucag	117
40	gu_ugguuuuag	113
41	gcaug_ag	112
42	gu_uuugagacag	109
1–42		213,943 (99.44%)

*Rank, the most to the least common consensus sequence. Donor-Acceptor (5S-3S), the intron sequences of the donor and acceptor sequences. Count, the count number of introns that have the indicated consensus sequence (N > 100). 1–42, the total number of introns in rank 1–42 is 213,949, which represents 99.44% of the total number of introns in humans*.

**Figure 1:**
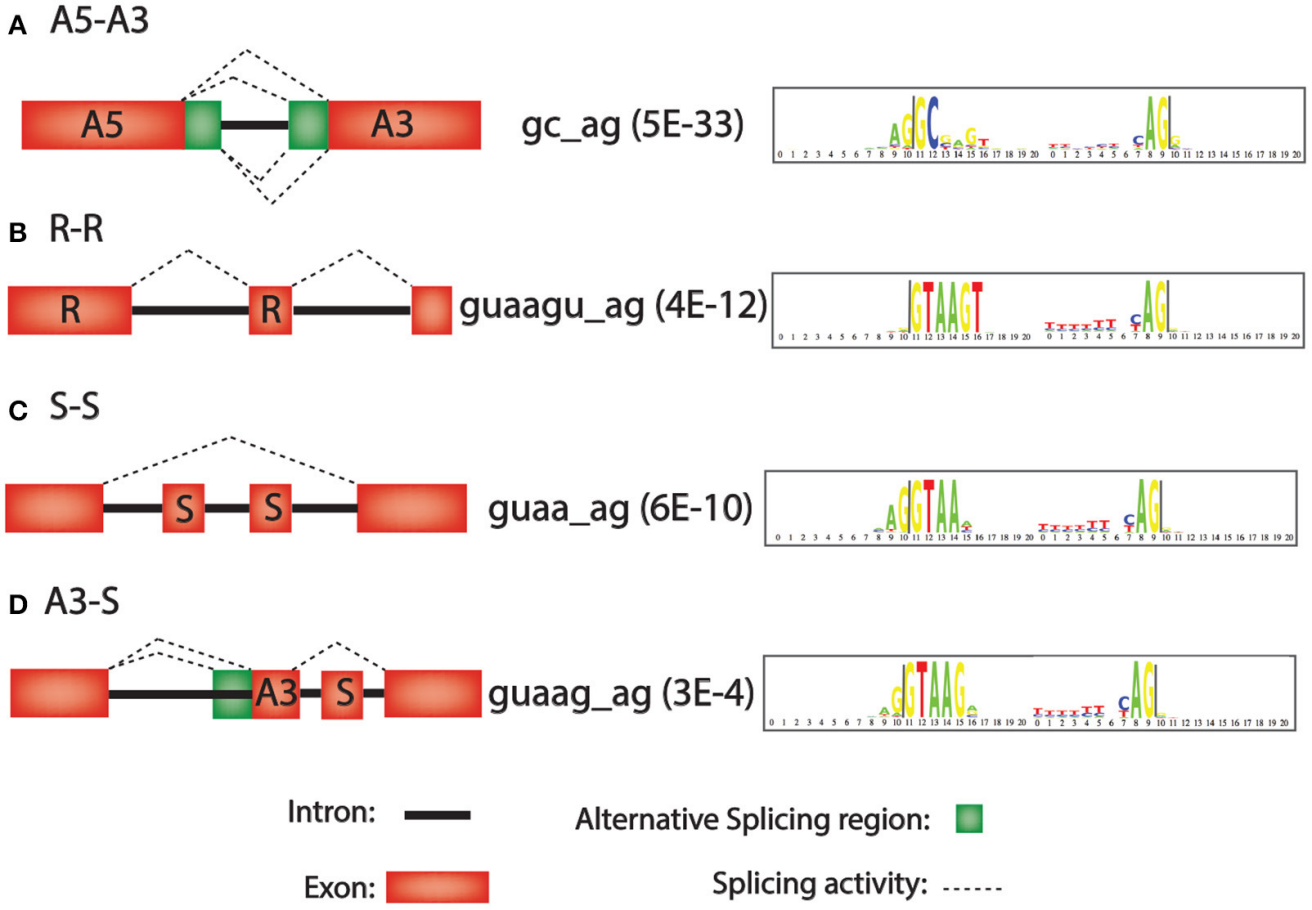
**Intron motifs that are over-represented in four representative alternative splicing classes**. Left, diagram of alternative splicing classes A5-A3, R-R, S-S, and A3-S. Middle, consensus sequence with most significant *p*-values for enrichment in this intron class (in parenthesis). Right, “logo plot” of consensus sequences. The larger the letter, the more frequent the nucleotide. **(A)** A3–A5 (Alternative 5′ splice site followed by an alternative 3′ splice site). **(B)** R-R (Retained exon followed by another retained exon). **(C)** S-S (Skipped exon followed by another skipped exon). **(D)** A3-S (Alternative 3′ splice site followed by a skipped exon).

Second, the 42 different paired consensus sequences in humans (Table 1) were analyzed individually to determine whether they are enriched or depleted for any of 36 types of introns (see Materials and Methods). The 36 types of introns are written in the form X_a_–X_b_, where X is one of the seven types of exon, and the X_a_ exon precedes the X_b_ exon in the same gene. For example, the class A5-A3 is an intron that is flanked by an upstream exon with an alternative 5′ splice site and a downstream exon with an alternative 3′ splice site. In Figure 1A, all 4 types of splicing, indicated with dashed lines, would generate introns in the A5-A3 class. Figures 1B–D show R-R, S-S, and A3-S classes of introns and the consensus sequences that are most significantly enriched for these classes of introns. Notice that there are only 36 possibly combinations for the 7 types of exons rather than 49 (i.e., 7^2^ = 49) because alternative poly A (APA) is never first (X_a_) and alternative promoter (APr) is never second (X_b_) in the X_a_-X_b_ nomenclature system.

In an attempt to concisely summarize our analyses, Table 2 shows an “alternative splicing code” for human introns in a format similar to the genetic code. The left column indicates the exon type upstream on the intron (APr, 3S, 5S, ME, R, and S) and the top row indicates the exon type downstream of the intron (3S, 5S, ME, R, S, and APA). The numbers in the table show the intron consensus sequences that are most significantly enriched (upper cell) and depleted (lower cell) for the indicated class of intron. Supplementary Figure 1 shows splicing diagrams of the 18 classes of introns that have consensus motifs that are over-represented for that intron class, and Supplementary Figure 2 shows splicing diagrams of the 16 classes of introns that have consensus motifs that are under-represented for that intron class. Notice that many of the classes of introns have more than one over-represented motif and more than one under-represented motif (Table 2), indicating that the alternative splicing code, much like the genetic code, is degenerate. Also, notice that almost half of the intron types are not associated with consensus sequences, such as A3–A5, possibly because macromolecular complexes presumably do not exist that recognize certain rare types of alternative RNA splicing event in humans.

**Table 2:** **The alternative mRNA splicing code for humans**.

**UP**	**A3**	**A5**	**ME**	**R**	**S**	**APA**
APr	11, 19, 26–28, 30	None	None	7, 10, 11, 14, 21, 22, 24, 30–33	7, 33, 42	None
	2, 3, 8			3, 5, 8	5	
A3	None	None	None	1, 4	2	None
				5	3	
A5	11, 14, 28, 30, 34, 42	4, 33	None	6, 10, 11, 14, 15, 19, 22, 25–28, 30–34	11, 12, 41, 42	None
	2, 3, 5, 7, 8, 10	3		1–3, 9	1, 8	
ME	None	None	None	None	None	None
				1		
R	1, 4, 32	None	None	2–4, 8, 9, 29, 37	1, 3, 42	1, 7
	5, 8, 11, 12, 17			7, 11, 16, 19, 21, 22, 24, 26, 27, 30–34, 42	11	4
S	None	13	None	6, 11, 20, 22, 41	3, 5, 13, 17, 18	3
		None		1, 4	4, 10, 14, 31	None

*The rows indicate the first exon (exon i) and the columns indicate the second exon (exon i+1) that flank an intron with the indicated consensus sequences. Listed are the consensus sequences that are significantly (P < 0.01) enriched (i.e., UP) for the indicated intron type (top numbers) and depleted for the indicated intron type (bottom numbers). The consensus sequences are listed alphabetically. Notice that many intron types are not significantly positively and/or negatively associated with a consensus sequence (none). A3, alternative 3′ splice site; A5, alternative 5′ splice site; ME, mutually exclusive exons; R, retained exons; S, skipped exons; APA, alternative polyA sites; APr, alternative promoter*.

Six of the 36 types of introns are enriched in the non-canonical core consensus sequence gc_ag, including APr-A3 (gc_ag, gcaag_ag, and gcaagu_ag), APr-R (gc_ag), A5-A3 (gc_ag and gcagg_ag), A5-R (gc_ag, gcaag_ag, gcaagu_ag, and gcagg_ag), A5-S (gcaug_ag), and S-R (gcaug_ag) (Table 2). However, the only intron type that is depleted in a non-canonical core consensus sequence is R-R, which is depleted for gc_ag, gcaag_ag, gcaagu_ag, and gcagg_ag (Table 2). We interpret this as indicating that many types of alternative splicing events utilize the non-canonical core consensus sequence gc_ag, but that invariant splicing almost always uses the canonical core consensus sequence gu_ag. These findings are consistent, with slight variations indicated below, in all 50 species studied. A complete list of paired consensus sequences is available upon request, as well as enrichment (up) and depletion (down) *p*-values for the 36 types of introns for each paired consensus sequence, for all 50 organisms' genomes that we analyzed (see Supplementary Table 2 for a partial list).

In our analyses of the 50 species, the most frequent intron class in most of the species is R-R, (e.g., 79% for *H. sapiens*, 29% for *M. musculis*, 62% for *D. melanogaster*, 80% for *C. elegans* and 91% for *A. thaliana*) which means a invariant exon is followed by another invariant exon (Figure 1B). The second most common intron class, in most of the 50 species analyzed, is S-S (e.g., 5% for *H. sapiens*, 22% for *M. musculus*, 4% for *D. melanogaster*, and 3% for *C. elegans*), which means that two consecutive exons are skipped, either together or individually, in mature RNA (Figure 1C). Other studies, have suggested that exon skipping is the most frequently occurring alternative splicing event. For example, it was found that over one third of exons can be skipped (~38%) (Ast, 2004; Sugnet et al., 2004). “Pathological” exon skipping is commonly seen in diseases with multiple disrupted alternative splicing events, especially in cancer (Watson and Watson, 2010).

**Figure 2:**
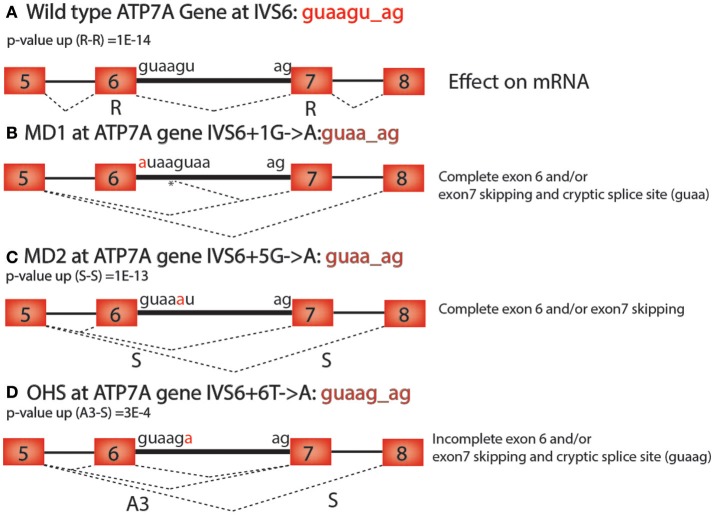
**ATP7A mutations in Menke's Disease. (A)** Wild type ATP7A intron 6 has the sequence guaagu_ag. The *p*-value (up) for this sequence in the Retained—Retained (R-R) class of introns is 2 × 10^−14^ (2E-14). **(B)** The MD1 mutation (IVS6+1G>A) in ATP7A causes complete exon 6 and/or exon 7 skipping and cryptic splice site usage at the 5th position in the intron at the sequence guaag_ag (^*^). **(C)** The MD2 mutation (IVS6+5G>A) in ATP7A causes complete exon 6 and/or exon 7 skipping and has the sequence guaa_ag. The *p*-value (up) for this sequence in the skipped—skipped (S-S) class of introns is 1 × 10^−13^ (1E-13). **(D)** The OHS mutation (IVS6+6T>A) in ATP7A is a weaker allele that causes incomplete exon 6 and/or exon 7 skipping and cryptic splice site usage at a second guaag_ag motif 50 base pairs downstream of the splice donor site. The *p*-value (up) for guaag_ag in the Alternative 3′ splice site—skipped class of introns is 3 × 10^−4^ (3E-4).

There are many practical uses for understanding the alternative RNA splicing code. For example, many diseases, including cancer, have mutations that cause changes in alternative RNA splicing that contribute to pathogenesis (Watson and Watson, 2010). It is estimated that at least 15–50% of mutations that cause human diseases affect splice-site selection (Wang and Cooper, 2007; Singh and Cooper, 2012). Here we show how the alternative RNA splicing code in Table 2 helps to interpret human genetic diseases that are caused by mutations near splice donor and acceptor sites that could not be adequately explained without this code. Using the databases of disease-causing mutations at spliced 3′ and 5′ splice sites, dbass5 and dbass3 (http://www.dbass.org.uk/dbass5/viewlist.aspx; Singh and Cooper, 2012), we analyzed all intron mutations at intron positions +3, +4, +5, and +6 (the first intron nucleotide at the splice donor is +1) and successfully correlated the alternative RNA splicing code to 96 different mutations in 56 genes (Supplementary Table 3). For example, Menkes disease (MD), which has several alleles in the ATP7A gene that are associated with alternative splicing defects, is a lethal disorder of copper metabolism that lead to severe neurological degeneration (Møller et al., 2000). Occipital horn syndrome (OHS) is a milder allelic form that is caused by partial loss of function of the ATP7A gene (Møller et al., 2000). Both MD and OHS are caused by mutations in the intronic sequences of the ATP7A gene, which encodes an ATPase that is responsible for copper efflux from cells (Figure 2A; Nissim-Rafinia and Kerem, 2002).

In the ATP7A gene, two splice-site mutations (IVS6+1G>A, IVS6+5G>A) for MD and one (IVS6+6T>A) for OHS were identified in a previous study (Figure 2; Møller et al., 2000). The main biological effect of the mutation in the first position of the splice donor site of intron 6 (gu to au) is cryptic downstream splice donor usage followed by exon 7 skipping (Figure 2B; Møller et al., 2000). Exon skipping and cryptic splice site activation are typical results of mutations in any of the four core consensus bases, gu_ag, and can be explained without the alternative splicing code. However, why the ATP7A mutation in position 5 of intron 6 (IVS6+5G>A) has such a severe effect on alternative splicing was previously not understood since this is outside of the canonical gu_ag consensus sequence (Figure 2C; Møller et al., 2000).

Using the alternative RNA splicing code, we can now better explain the alternative splicing phenotypes caused by the mutations the 5th position of the 5′ splice site of intron 6 of ATP7A. The wild-type sequence guaagu_ag corresponds to a paired consensus sequence that is over-represented for R-R, which means that there is little or no alternative splicing in the wild-type ATP7A gene for this intron (Figure 1A). However, the 5th position mutation (Figure 2C) corresponds to the guaa_ag paired consensus sequence that is over-represented for the intron class S-S (Figure 1C). Therefore, the alternative RNA splicing code helps explain why two adjacent exons, exons 6 and 7, are skipped as the result of the mutation in the 5th position (Figure 2C). A similar argument can be made for the milder ATP7A mutation in OHS, (IVS6+6T>A), which leads to a motif change to guaag_ag, which is an over-represented motif for the intron class A3-S, and leads to incomplete exon 6 and/or exon 7 skipping and cryptic splice site usage 50 nucleotides downstream of the normal 5′ splice site in intron 6, at a second guaag_ag sequence (Figure 2D). In the OHS allele, exon 6 becomes an A3 exon because the 5′ splice site of exon 5 can join with the normal 3′ splice site or exon 6 or the alternative 3′ splice sites of exon 7 or exon 8 (Figure 2D).

We note that the above analysis for ATP7A intron 6 is an over simplification of what is required to predict the effect of an intron mutation because multiple consensus sequences are often enriched or depleted in several of the 36 types on introns. For example, the wild type ATP7A intron 6 consensus sequence, guaagu_ag, corresponds to a consensus sequence that is enriched for R-R, R-S, and S-APA. Therefore, in order to predict the outcome of a mutation in a consensus sequence, one must determine which intron classes are uniquely enriched when a mutation is present that was not enriched in the wild-type sequence. The sixth position mutation in OHS has the intron sequence guaag_ag which is enriched in A3-S and R-R. This might explain why both A3-S and R-R splicing events are induced by the OHS mutation (Figure 2D). Similarly, the fifth position mutation in MD2 has the sequence guaa_ag, which is only enriched in the intron type S-S. This might explain why S-S splicing events are induced by the MD2 mutation (Figure 2C). The alternative RNA splicing code can also be used to explain +3 to +6 intron mutations in neurofibromatosis type 1 (NF1), one of the most prevalent inherited disorders in human (Hastings and Krainer, 2001), beta thalassemia (HBB) (Felber et al., 1982), and many other human diseases.

In addition to the canonical splicing pathway, which uses the gu_ag consensus sequence, there are non-canonical (a.k.a., minor) splicing pathways that sometimes do not use the gu_ag consensus (Padgett, 2012). The canonical splicing pathway generally uses the U1 and U2 small RNAs in their splicing mechanism, always at gu_ag introns, while the non-canonical pathway uses U11 and U12 small RNAs, at both gu_ag and au_ac introns. The U12-like introns also have several conserved nucleotides that flank the splice donor and splice acceptor sequences (Padgett, 2012). When we searched for U12-like consensus sequences in the lists of intron consensus sequences, we found that human and mouse share the top three U12-like sequence matches: (1) guauccuuu_ag (Rank 29, Table 1), (2) auauccuu_ac (Rank 37, Table 1) and (3) guauccuu_ag (Rank 36, Table 1). The U12-like motif guauccuuu_ag is also the best match with the U12-like splicing pathway in *A. thaliana* (Supplementary Table 2). Curiously, both *D. melanogaster* and *C. elegans* have the weakest matches to the U12-like splicing sequence, gugggu_cag, and guucguuuuu_uuucag, respectively, even though they are presumably evolutionarily closer to humans than plants (Supplementary Table 2).

**Figure 3:**
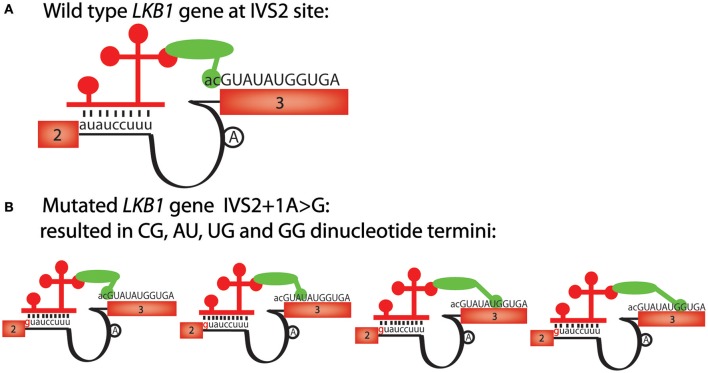
**The alternative mRNA splicing code predicts the effects of a U12-type intron mutation, IVS2+1A>G, in the LKB1 gene. (A)** Schematic of human LKB1 wild-type gene sequence, has a “auauccuu_ac” U12-like intron consensus sequence. Exon numbers are shown in boxes, sequences belong to exons are uppercase; lines represent introns, sequences belonging to introns are lowercase. The 5′ and 3′ splice site recognition machinery of the U12 splicesosome complex are shown schematically. The lariat site is shown as an “A” in a black circle. The red bars represent U11 and the green oval is a simplified version of multiple interacting factors involved in the splicing events. **(B)** The mutation in Peutz-Jegher's syndrome (IVS2+1A>G) changes the U12-like consensus sequence from auauccuu_ac to guauccuu_ag. However, since no “ag” is detected at the 3′ splice site, cg, au, ug and gg become alternative dinucleotide termini. The red bars represent U11 and the green oval is a simplified version of multiple interacting factors involved in the splicing events.

As we showed with mutations that affect the major splicing machinery, mutations that affect the minor splicing machinery can also be better interpreted with the paired consensus sequence motifs that we identified. One example involves a tumor suppressor gene, LKB1, whose splice acceptor mutation in the second intron is thought to cause Peutz-Jeghers syndrome (PJS) (Hastings et al., 2005). This mutation changes the splice junction sequence from auauccuu_ac to guauccuu_ac, and causes aberrant splicing, even though the mutation is changing a non-canonical “au” splice donor to a canonical “gu” splice donor (Figure 3A). Perusing the alternative RNA splicing code, we noticed that the wild-type LKB1, auauccuu_ac, is present, but the sequence found in PJS, guauccuu_ac, is not present on the paired RNA splicing consensus sequence table in humans (Table 1). Therefore, even though the consensus sequence table indicates that the splice donor sequence guauccuu is a good minor splice donor sequence, the paired-sequence analyses indicate that the “gu” core splice donor sequence must be paired with another canonical splice acceptor sequence, “ag,” even in U12-type introns. In other words, our analyses suggest that there are at least two distinct classes of U12-type introns in humans; one with the core sequence gu_ag and the other with au_ac, and the machinery that recognizes the two ends of the introns in the U12-type splicesosomes cannot be swapped. This hypothesis might also help explain the unusual splicing reactions at the 3′ splice site to be multiple cryptic dinucleotide termini (such as cg, au, ug, and gg) observed from different patients since no “ag” is present in vicinity of the splice acceptor site (Figure 3B; Hastings et al., 2005).

Here, we admit that interaction through direct pairing of both the 5′ and 3′ splice-site recognition complexes is one of the possible explanations that affect splicing events. In our hypothesis for developing the alternative splicing code, we emphasized that the 5′ and 3′ splice sites should be considered as a pair. However, mostly these sites can be recognized by independent complexes, 5′ splice sites by U1 and 3′ splice sites by U2AF in the first step of canonical splicing. In addition, in most of the consensus sequences we identified, the 3′ splice sites are far less variable than the 5′ splice sites. Other possibilities that could explain the alteration of splicing events which need to be further investigated include interaction of splicing enhancers or silencers.

**Figure 4:**
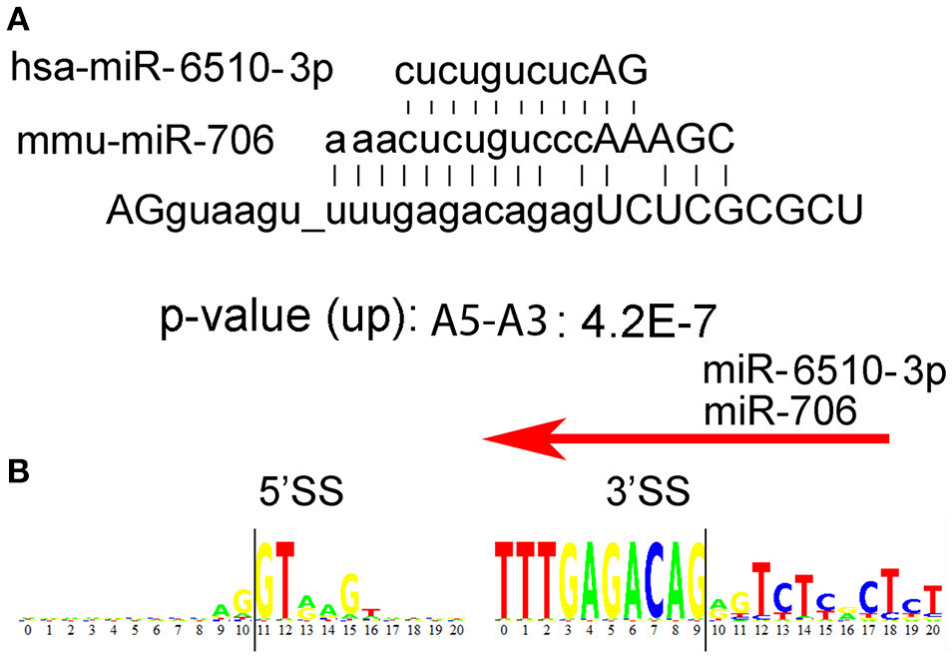
**Possible regulation of alternative mRNA splicing by miRs. (A)** We used the Mirbase database http://www.mirbase.org to search for miRNA sequences that might bind to the long human intron motifs, AGguaagu_uuugagacagagUCUCGCGCU. Human hs-miR-6510-3p as well as mouse mmu-miR-706n are excellent matches. **(B)** Donor 5′ and acceptor 3′ consensus motif for the consensus sequence AGguaagu_uuugagacagagUCUCGCGCU in humans. The intron begins at position 11 on the left and ends at position 9 on the right.

In addition to the long consensus sequences identified in the U12-type non-canonical introns by rank-order analyses (Table 1), we found several uncommonly long non-U12-type consensus sequences (>8 nucleotides) in humans that are also conserved in mouse, but not *C. elegans, D. melanogaster*, and *A. thaliana*. Splice junctions that are targeted by antisense oligonucleotides can often block alternative splicing, such as “morpholino” oligonucleotides that are used to inhibit splicing (Morcos, 2007). Therefore, one possibility is that the long consensus sequences might be targets for long-noncoding RNA or RNA-binding proteins that regulate alternative RNA splicing in these introns. To test this idea, we used the Mirbase database (http://www.mirbase.org) to search for complimentary miRNA sequences for the long human and mouse alternative mRNA splicing motifs. We found that the long paired alternative RNA splicing motif gu_uuugagacag (Rank 42, Table 1) is an excellent match with human hs-miR-6510-3p as well as mouse mmu-miR-706 (Figure 4A). This bioinformatics result suggests that this uncommonly long splice site consensus sequence might be negatively regulated by one of these microRNAs, but this hypothesis requires genetic validation (Figure 4B).

**Figure 5:**
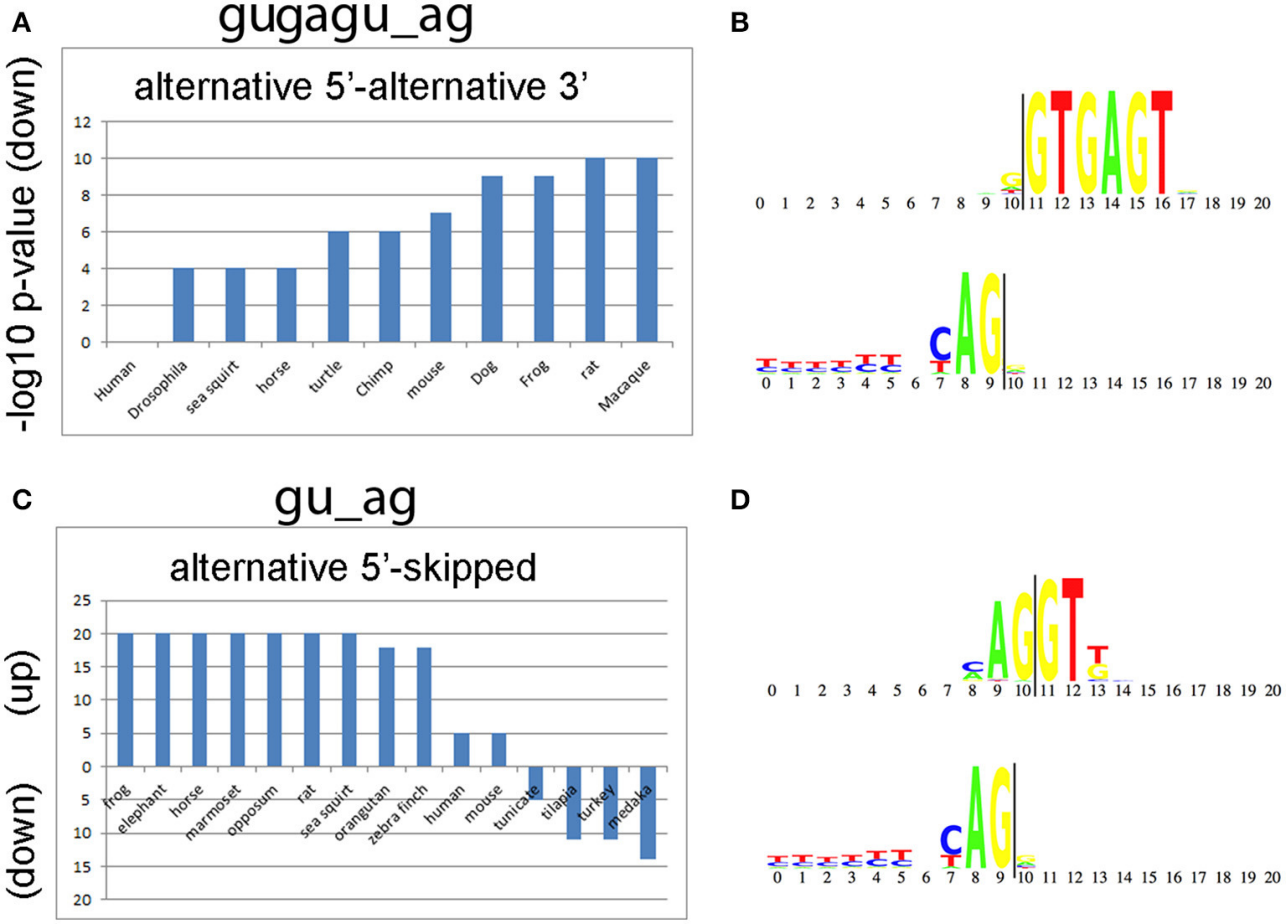
**Conserved splicing motifs in 50 species. (A)** The alternative 5′ splice site—alternative 3′ splice site (A5-A3) class is under represented for the consensus sequence gugagu_ag in 10 of the 50 species analyzed. **(B)** Donor and acceptor motif structure for gugagu_ag class. The splice donor (gu) starts at 11 and the splice acceptor (ag) ends at position 9 (vertical lines). **(C)** The alternative 5′ splice site—skipped exon (A5-S) class is enriched for the consensus sequence gu_ag in 11 of the 50 species and depleted in 4 of the 50 species analyzed. The y-axis shows the –log10 *p*-values for gu_ag introns enrichment (up) or de-enrichment (down) with the A5-S class. **(D)** Donor and acceptor motif structure for gu_ag class.

We also compared alternative splicing consensus sequences among the 50 species to determine whether they are evolutionarily conserved and whether they are enriched in the same classes of introns. The top two consensus sequences that are shared by the greatest number of species are gugagu_ag and gu_ag (Figure 5). The motif gugagu_ag is enriched for the intron class A5-A3 in 10 of the 50 species (Figure 5A), and the motif gu_ag is enriched in the intron class A5-S in 11 of the 50 species and depleted in the intron class A5-S in 4 of the 50 species (Figure 5B). Humans and mouse share 80% of all alternative RNA splicing motifs. When we looked for a possible reason why *D. melanogaster, C. elegans* and *A. thaliana* share only small portion of significant motifs with human (14, 26, and 29%), we found that, although the canonical sequence gu_ag is the most highly conserved (98%), the third base after the splicing donor “gu” varies among taxa. The base adenine was hardly ever observed in the third position of the intron donor sequence in *D. melanogaster* or *C. elegans* (<1%), while adenine is the most common nucleotide in the third position in the splice donor, i.e., gua_ag, for both human (58.4%) and mouse (58.3%).

**Figure 6:**
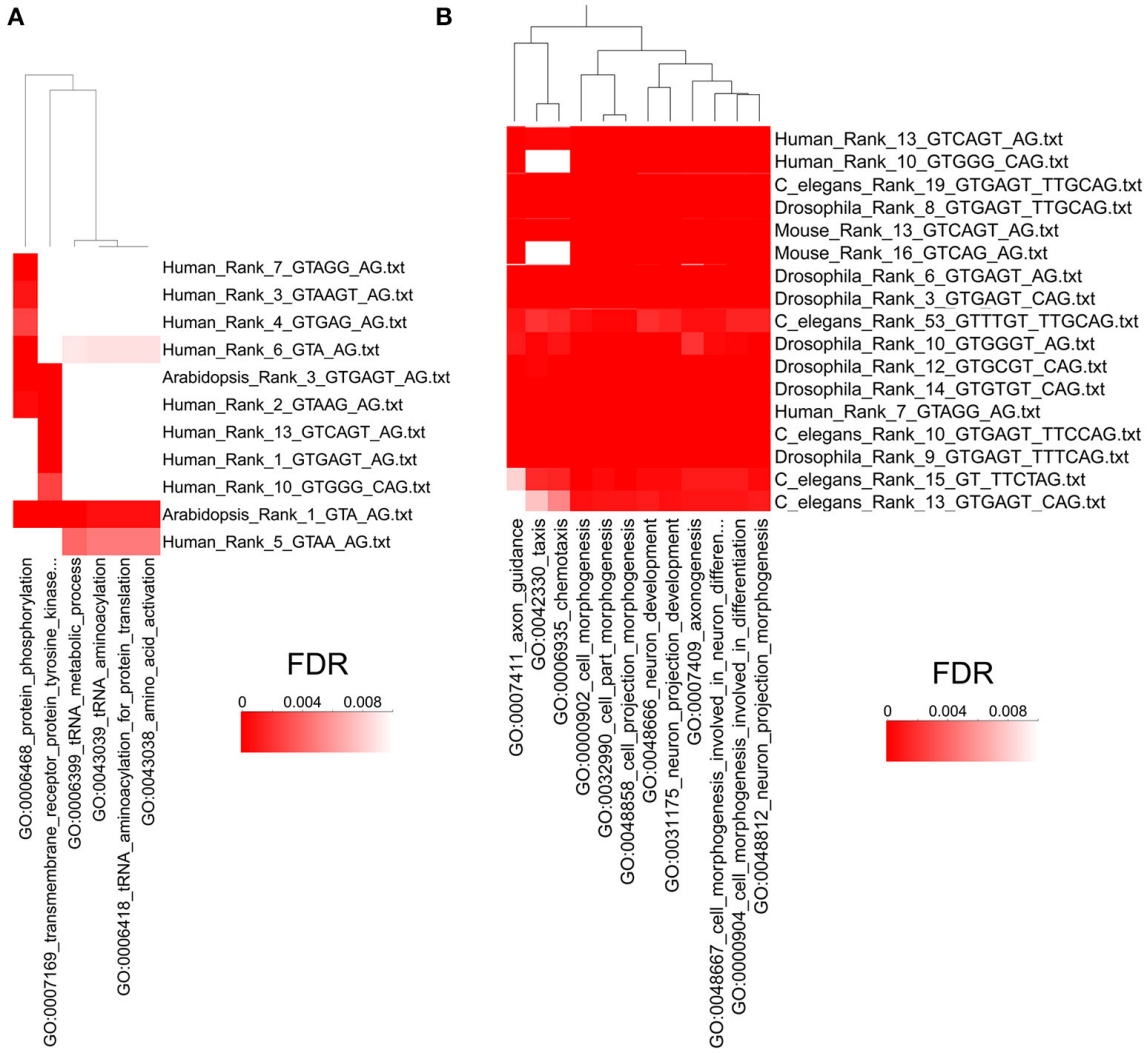
**High Throughput GOMiner Analysis of Alternative Splicing Consensus Sequences. (A)** HTGM analysis of human and *A. thaliana* consensus sequences shows that phosphorylation and metabolic processes share evolutionarily conserved alternative splicing consensus sequences. The *p*-values of the GO classifications are indicated by a heat map where the darkest red has an FDR *p*-value of 0.001 or less. The consensus sequences are shown on the right and “Rank” refers to the rank order of introns that have that consensus sequence, where 1 is the most common sequence. The GO categories that are shared are shown on the bottom and the clustering dendrogram is shown on the top. **(B)** HTGM analysis of *H. sapiens, M. musculus, D. melanogaster*, and *C. elegans* shows that neurogenesis and developmental processes share evolutionarily conserved alternative splicing consensus sequences. Figure 6B represents only a small portion of the HTGM analyses of these four animals. See **S**upplementary Figure 3 for the full HTGM heat map diagram of the four animals analyzed.

Next, we used the program High-Throughput GoMiner (HTGM) (Zeeberg et al., 2005) to analyze and interpret the functional significance of conserved gene sets that have at least one intron with identical paired consensus sequences between humans and *A. thaliana*. In this analysis, significant Gene Ontology (GO) categories were determined for genes with a single particular type of paired consensus sequence in an intron. Not surprisingly, since they are separated by hundreds of millions of years of evolution, not many paired alternative splicing motifs are conserved between humans and plants. However, the paired motif gugagu_ag is the third most common motif in *A. thaliana* (i.e., Rank 3) and the most common motif in humans (Rank 1). Similarly, gua_ag is the most common motif in *A. thaliana* and the 6th most common motif in humans. Genes that have these two sets of motifs in *A. thaliana* and humans cluster together in the HTGM analysis with the GO categories “protein phosphorylation,” “protein tyrosine kinase,” “tRNA metabolic processes,” “tRNA aminoacetylation,” and “amino acid activation” (Figure 6A). The identification of GO categories in protein phosphorylation is interesting because species-specific alternative exons were analyzed in a recent study, and the two most enriched keywords are “alternative splicing,” which reflects the abundant alternative RNA splicing in this set of genes, and “phosphorylation” (Merkin et al., 2012). Alternative RNA splicing is used to alter protein phosphorylation, which can alter protein stability, subcellular localization, and activity (Merkin et al., 2012). Our results suggest that using alternative RNA splicing to regulate phosphorylation of proteins might be an evolutionarily conserved mechanism in plants and humans and is regulated by these two shared alternative RNA splicing motifs (Figure 6A).

We also did HTGM analyses of the gene sets with identical paired alternative RNA splicing sequences in *H. sapiens, M. musculis, C. elegans*, and Drosophila, but excluding *A. thaliana* because it is too divergent from the other species. Multiple-species cluster analyses of Gene Ontology categories determined by HTGM indicates that “cell morphogenesis” and “cell development” and related GO categories are conserved across the four animal species for multiple similar consensus sequences (Figure 6B; for a full HTGM analysis of the four animals see Supplementary Figure 3). Our HTGM result in the four animal species is consistent with previous studies demonstrating alternative splicing is often evolutionarily conserved in a tissue and developmental stage-specific manner (Chen et al., 2012; Merkin et al., 2012).

Finally, we wanted to determine whether genes with multiple introns tend to have the same or different intron consensus sequences. If, as we hypothesize, the 36 types of introns in the alternative splicing code table (Table 2) utilize many different types of splicesosomes, then it is possible that genes with multiple introns will utilize as few different types of splicesomes as possible. It might not be practical for different types of splicesomes to splice each of the different introns in a gene. Rather, since RNA splicing occurs concurrently with transcription, it might be more efficient for a particular type of spicesosome to move on to the next intron once it has completed the splicing reaction of the upstream intron.

**Figure 7:**
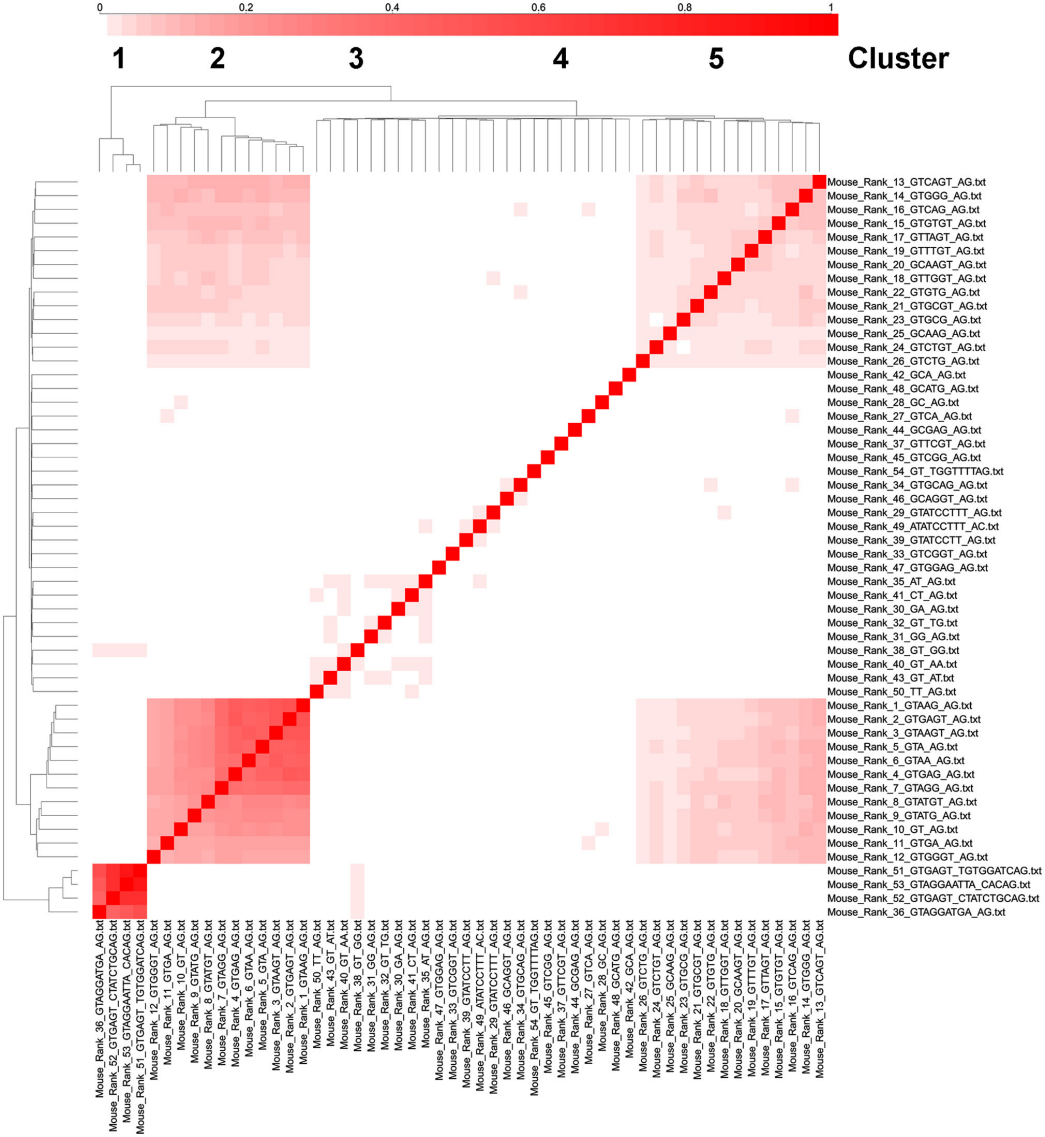
**Cluster analysis of intron consensus sequences in the same gene in mouse**. The 53 ranked intron consensus sequences from Mus musculus were clustered based on whether they were in the same gene as the same or another ranked intron consensus sequence. The diagonal boxes represent unity (i.e., a gene with a rank 1 intron always has a rank 1 intron, etc.). Notice that ranks 36,51,52, and 53 cluster together (these are all U12 type introns), as well as ranks 1–12 (the most common consensus sequences), 13–26 (the next most common consensus sequences) and ranks 27–50 (except rank 36 which is a U12-type intron in the first cluster).

The most significant cluster of intron types with genes with multiple introns corresponds to U12-like introns (rank 36, 51–52 in mouse) and the second most significant cluster corresponds to U2-like introns that correspond to the top 12 ranked consensus sequences (Figure 7). Introns in ranks 13–26 form a third cluster and introns in ranks 27–50 (excluding rank 36, which is a U12-type intron) form a fourth cluster, but this is not well segregated from the third cluster (Figure 7). We interpret this as suggesting, using the logic in the previous paragraph, that there might be as many as 3 or 4 different types of splicesosomes in the mouse. We performed similar cluster analyses in Arabidopsis, *C. elegans*, Drosophila, and humans and identified 3 or 4 similar clusters of intron consensus sequences in genes with multiple introns (Supplementary Figures 4–7). In humans, rank 1–42 introns all have remarkably similar distributions in genes with 1–14 introns. For example, over 50% of all genes have 6–10 introns, and over 50% of all the rank 1–42 introns are in genes with 6–10 introns (Supplementary Figure 8).

## Discussion

The alternative mRNA splicing code in Table 2 provides a new toolkit for characterizing cis-acting sequences that are important for generating the enormous diversity of processed mRNAs in animals and plants. Other groups have attempted to decipher the alternative mRNA splicing code by characterizing the binding sites for a large number of RNA binding proteins that are known to affect tissue-specific alternative mRNA splicing, such as MBNL, PTB, RBFOX, STAR, and TIA families of splicing factors (Ladd and Cooper, 2002). The research group of Xiong et al. (2015) have extracted human DNA sequences as input and used a Bayesian model to predict the percentage of exons that are spliced in (PSI) based on sequences within the transcripts. For example, they found among intronic variants that are known to cause disease alter splicing nine times as often as the common variant when they are more than 30 nucleotides away from any splice site. We believe that our study complements the study by Xiong et al. because their program can be used to calculate PSI-values, whereas, our method will help to predict the type of alternative splicing that SNPs with altered PSI-values produce. We did not investigate branchpoints in our paper, but Mercer et al. (2015) analyzed RNA-seq data with novel bioinformatics methods to identify 60,000 high-confidence human branchpoints.

Combining our alternative mRNA splicing consensus sequence information with the RNA-binding protein datasets should allow the construction of better RNA splicing maps (Witten and Ule, 2011) that can be used to better understand the mechanism of tissue-specific alternative mRNA splicing events. The alternative RNA splicing code can also be used to better understand how human germline disease mutations and somatic mutations in cancer affect alternative RNA splicing and lead to disease etiology. Future biochemical experiments are needed to test the hypothesis that the many classes of paired alternative RNA splicing events in humans with paired consensus sequences have unique macromolecular complexes that regulate RNA maturation. Future bioinformatics analyses are needed to predict how a particular splice site mutation in any of the first or last few nucleotides in an intron precisely affects alternative splicing. The alternative splicing code should help inform both of these endeavors.

## Materials and methods

### SnpEff analyses and identification of paired consensus sequences

#### Pre-filtering

For all genes in each analyzed genome, protein-coding transcripts are curated and filtered out if putative annotation errors or inconsistencies are found in the reference genome.

#### Intron characterization

For all protein coding transcripts, each exon is characterized by splicing type: retained (R), skipped (S), alternative 3′ splice site (A3), alternative 5′ splice site (A5), mutually exclusive (ME), alternative promoter (APr), and alternative poly-A (APA). This characterization is performed as defined in the text. Each intron is labeled according to its flanking exons, for instance, an intron flanked by a *retained* exon and a *skipped* exon, is labeled as *retained-skipped (R-S)*.

#### Splice site sequence analysis

Unique introns, defined by their genomic coordinates, are analyzed using their splice site donor and acceptor sequences using up to 10 bases on each side of the intron. Splice site donor and acceptor sequences are added to a quaternary tree (a tree of sequences of A, C, G, T), according to their DNA sequence. These quaternary trees are paired for splice site donors and acceptor sequences. Probabilities and entropies are calculated on each tree branch of these quaternary trees, for all branches having at least 100 sequences. Pairs of donor acceptor sequences are selected from the quaternary tree branches in the 95% probability percentile and the 5% entropy percentile, these are selected as highly conserved. Fisher exact test is calculated for each intron category in each conserved splice site sequence donor-acceptor, over represented (*p*-value upper tail) or under-represented (*p*-value lower tail) categories are reported if their *p*-values are <0.001.

#### Branch splice sequence analysis

Intron sequences near the 3 prime end of the intron, up to 60 bases, are scanned for matching U12 position weighted matrices (PWMs). The best match in each intron is selected and the empirical probability distribution is calculated, top 5% scores are selected as significantly matching a U12 motif. Expected number of matching introns for each intron category is calculated and intron categories having an unexpected number of observed/expected matches are selected as significant. The number of introns matching a top 5% score, as well as observed/expected ratios are reported.

#### High throughput GoMiner analyses

GoMiner (Zeeberg et al., 2003) is a tool for biological interpretation of 'omic' data, including data from gene expression microarrays and state of the art sequencing technologies. It leverages the Gene Ontology (GO) to identify “biological processes,” “molecular functions,” and “cellular components” represented in a list of genes. High-Throughput GoMiner (HTGM) (Zeeberg et al., 2005), which was used for many of the analyses reported here, is an enhancement of GoMiner that efficiently performs the computationally-challenging task of automated batch processing of an arbitrary number of such gene lists. A GO category is *enriched* if the number of changed genes that HTGM assigned to it is statistically significantly greater than the number expected by chance. A category is considered *significant* if its Fisher's Exact *p*-value and its false discovery rate (FDR) are both less than or equal to a user-selected threshold (typically 0.10; on rare occasion, the *p*-value can exceed the threshold although the FDR is below the threshold, and we usually want to reject such instances). See Zeeberg et al. (2003, 2005) for detailed discussions of GoMiner and HTGM, including calculations of statistical significance. HTGM runs were performed separately for each of the several species studies. Parameter values for each run are summarized in Supplementary Table S4. When results from two or more studies [i.e., HTGM genes vs. categories clustered image map (CIM)] were to be combined, In-house R code was used to combine the individual CIM files into a composite file, and to render the CIM images.

## Author contributions

DR is the PI of the lab and directed the analyses and the writing of the manuscript. WQ helped write the paper, made the figures, and performed the human SNP analyses. PC wrote the software and came up with the original idea, BZ did the multiple species GO analyses.

### Conflict of interest statement

The authors declare that the research was conducted in the absence of any commercial or financial relationships that could be construed as a potential conflict of interest.
