# Supplementary material for: A Bioinformatics-Based Alternative mRNA Splicing Code that May Explain Some Disease Mutations Is Conserved in Animals
Source: Front Genet. 2017 Apr 11;8:38. doi: 10.3389/fgene.2017.00038 (PMC5387049; doi:10.3389/fgene.2017.00038)

Supplement Figures and Legends

Supplement Fig. 1. *Motifs that are over-represented in alternative splicing classes*.

Left, diagram of alternative splicing class. **Middle**, consensus sequence with most significant p-value (in parenthesis). Right, “logo plot” of consensus sequence. The larger the letter, the more frequent the nucleotide.

a, A3-APA (Alternative 3’ splice site followed by an alternative poly A adenylation site).

b, A3-R (Alternative 3’ splice site followed by a retained exon).

c, A3-S (Alternative 3’ splice site followed by a skipped exon).

d, A5-A3 (Alternative 5’ splice site followed by a alternative 3’ splice site).

e, A5-A5 (Alternative 5’ splice site followed by another alternative 5’ splice site) .

f, A5-R (Alternative 5’ splice site followed by a retained exon).

g. A5-S (Alternative 5’ splice site followed by a skipped exon).

h, S-R (Skipped exon followed by a retained exon).

i, Apr-A3 (Alternative promoter site followed by an alternative 3’ splice site).

j, S-APA (Skipped exon followed by an alternative ploy A adenylation site).

k, S-S (Skipped exon followed by another skipped exon).

l, S-A5 (Skipped exon followed by an alternative 5’ splice site).

m, Apr-R (Alternative promoter site followed by a retained exon).

n, APr-S (Alternative promoter site followed by a skipped exon) .

o, R-A3 (Retained exon followed by an alternative 3' splice site).

p, R-APA (Retained exon followed by an alternative ploy A adenylation site).

q, R-R (Retained exon followed by another retained exon).

r, R-S (Retained exon followed by a skipped exon).

Supplement Fig. 2. *Motifs that are under-represented in alternative splicing classes.*

Left, diagram of alternative splicing class. Middle, consensus sequence with most significant p-value (in parenthesis). Right, schematic plot of consensus sequence. Uppercase represents exon region and lowercase represents intron region. The larger the letter, the more frequent the nucleotide.

a, A3-R (Alternative 3' splice site followed by a retained exon).

b, A3-S (Alternative 3' splice site followed by a skipped exon).

c, A5-A3 (Alternative 5' splice site followed by an alternative 3' splice site).

d, A5-A5 (Alternative 5' splice site followed by another althernative 5' splice site).

e, A5-R (Alternative 5' splice site followed by a retained exon).

f, A5-S (Alternative 5' splice site followed by a skipped exon).

g, APr-A3 (Alternative promoter site followed by alternative 3' splice site).

h, APr-R (Alternative promoter site followed by a retained exon).

i, APr-S (Alternative promoter site followed by a skipped exon).

j, ME-R (Mutually Exclusive exons followed by a retained exon).

k, R-A3 (Retained exon followed by an alternative 3’ splice site).

l, R-APA (Retained exon followed by an alternative poly A adenylation).

m, R-R (Retained exon followed by another retained exon).

n, R-S (Retained exon followed by a skipped exon).

o, S-R (Skipped exon followed by a retained exon).

p, S-S (Skipped exon followed by another skipped exon).

Supplement Fig. 3. Full HTGM (High Throughput GO Miner) analysis diagram of H. sapiens, M. musculus, D. melanogaster, and C. elegans. See **Figure 6** for a smaller version of this diagram.

Supplement Fig. 4. Cluster analysis of intron consensus sequences in Arabidopsis in genes with more than one intron.

Supplement Fig. 5. Cluster analysis of intron consensus sequences in C. elegans in genes with more than one intron.

Supplement Fig. 6. Cluster analysis of intron consensus sequences in Drosophila in genes with more than one intron.

Supplement Fig. 7. Cluster analysis of intron consensus sequences in humans in genes with more than one intron.

Supplement Fig. 8. Percent introns of rank 1-42 in genes with 1-14 introns in humans.

a. The average percentage for all introns in ranks 1-42 is shown on the y-axis and the number of introns in a gene is shown on the x-axis. The equation is the best fit binomial equation and the correlation coefficient (R2) is shown.
b. The percentage of introns with Rank 1 in genes with 1-14 introns.
c. The percentage of introns with Rank 2 in genes with 1-14 introns.
d. The percentage of introns with Rank 42 in genes with 1-14 introns.

Supplementary Figure 1

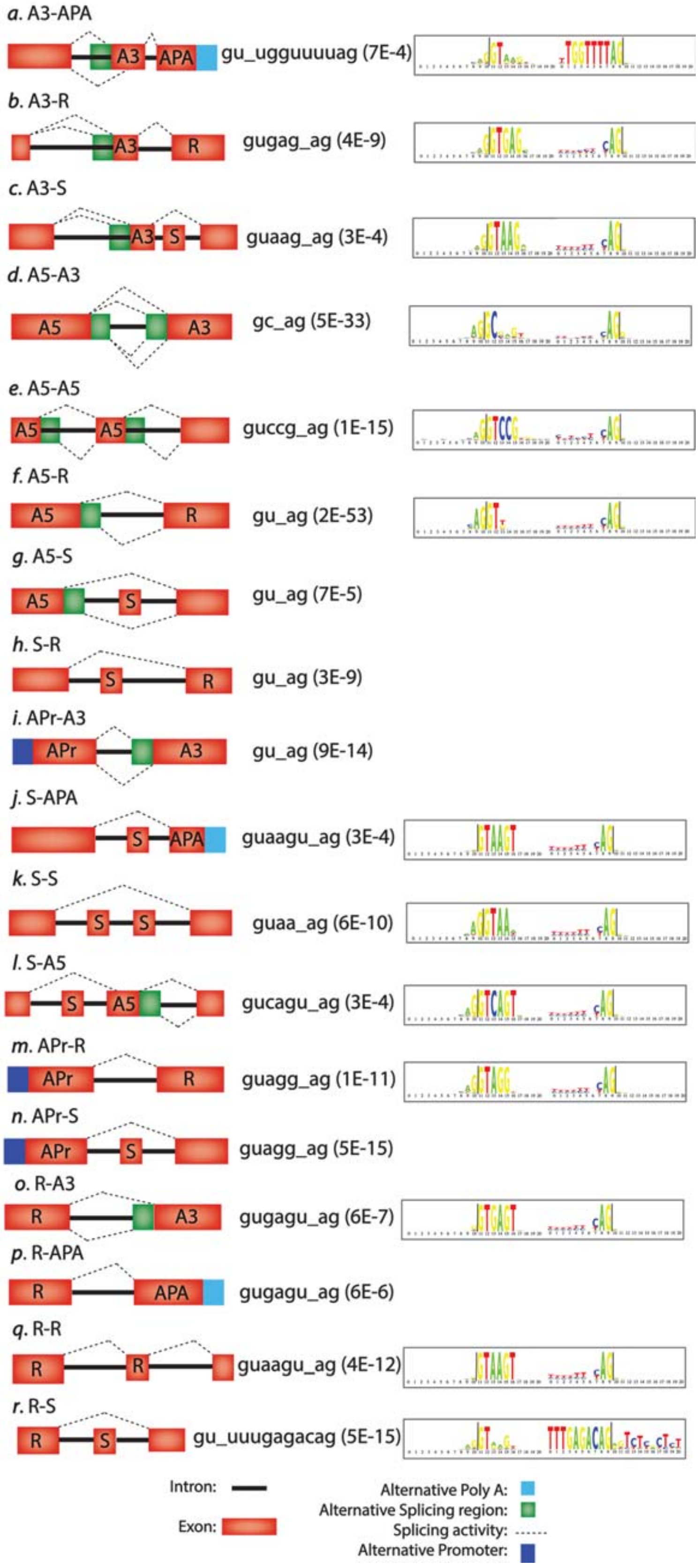

Supplementary Figure 2

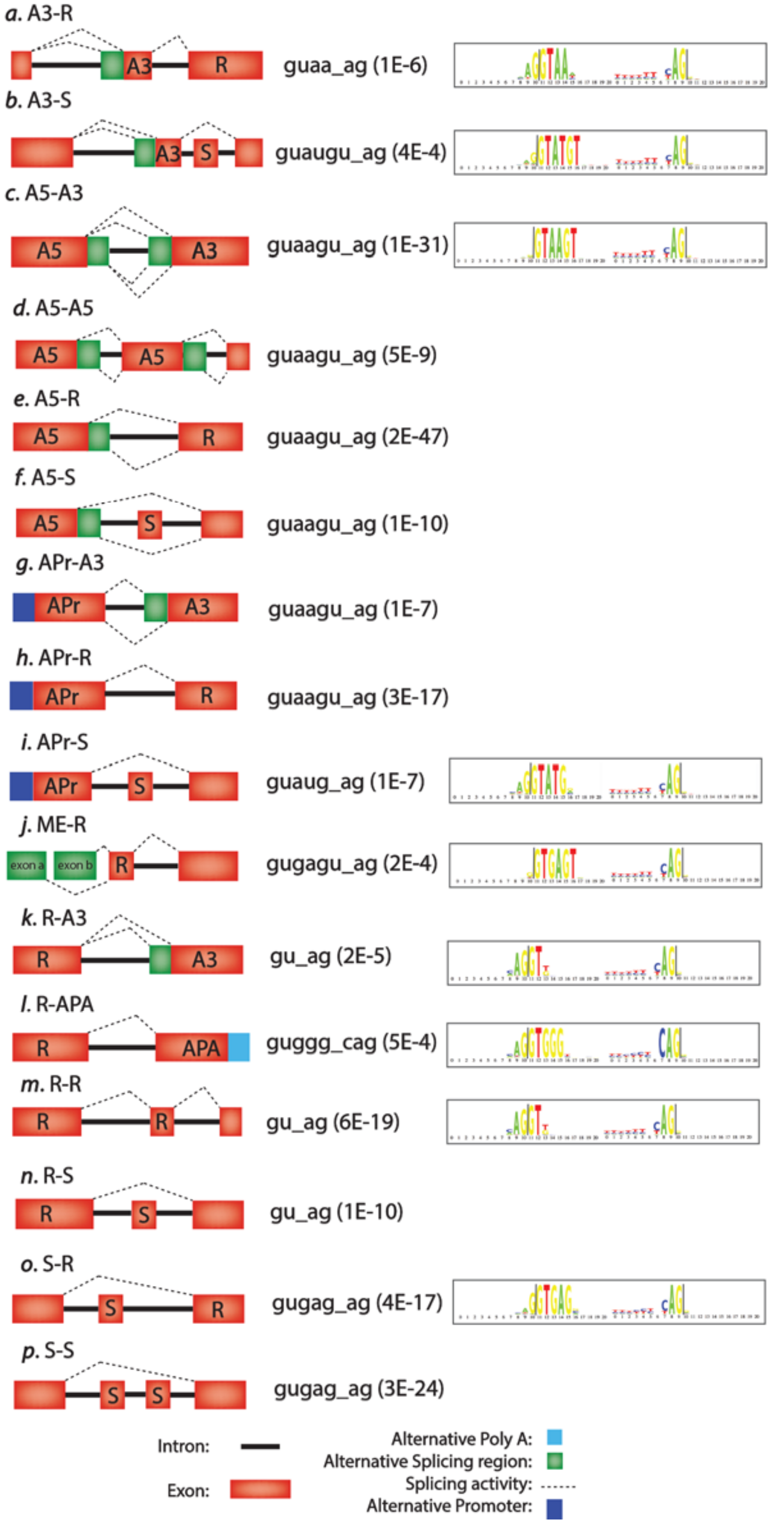

Supplementary Figure 3

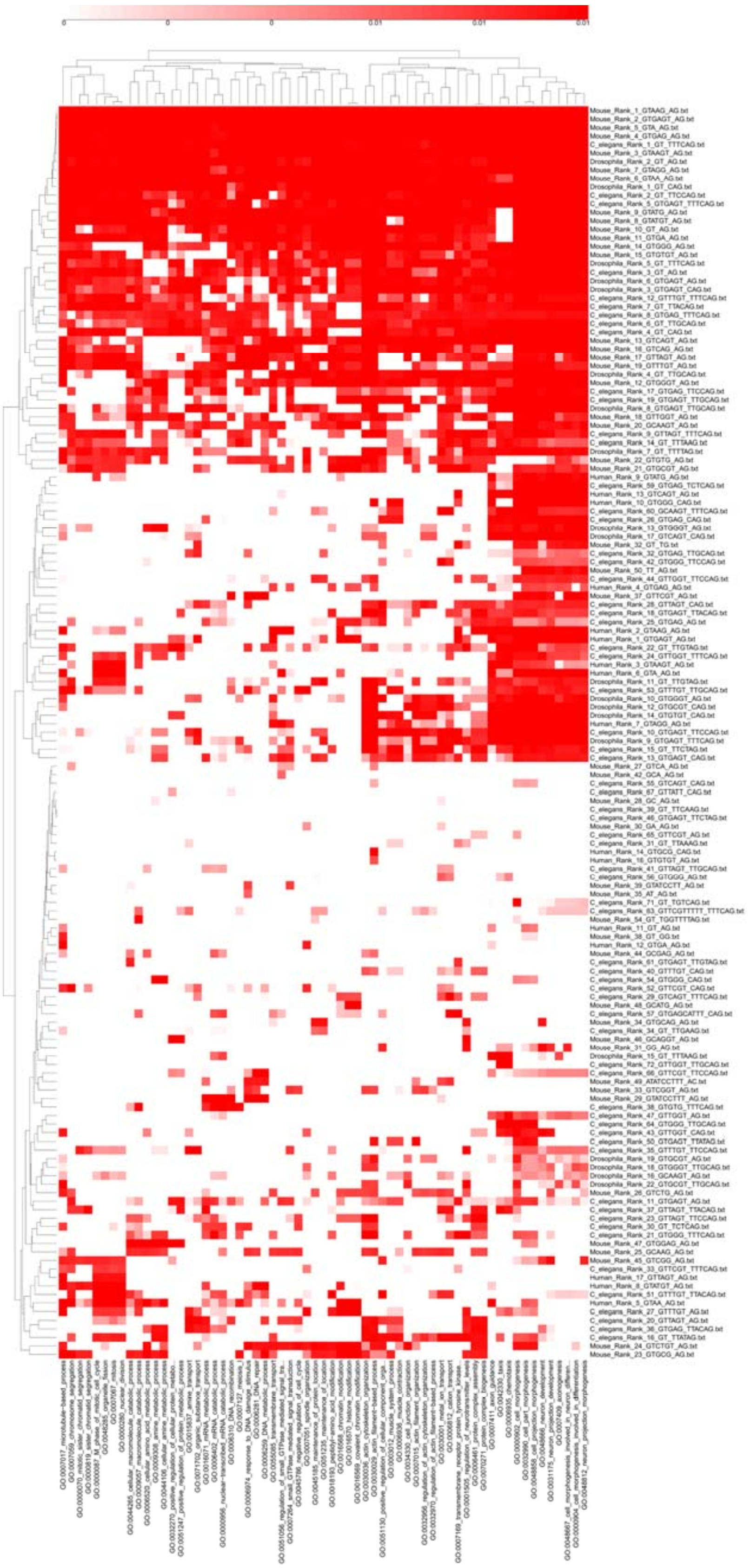

Supplementary Figure 4

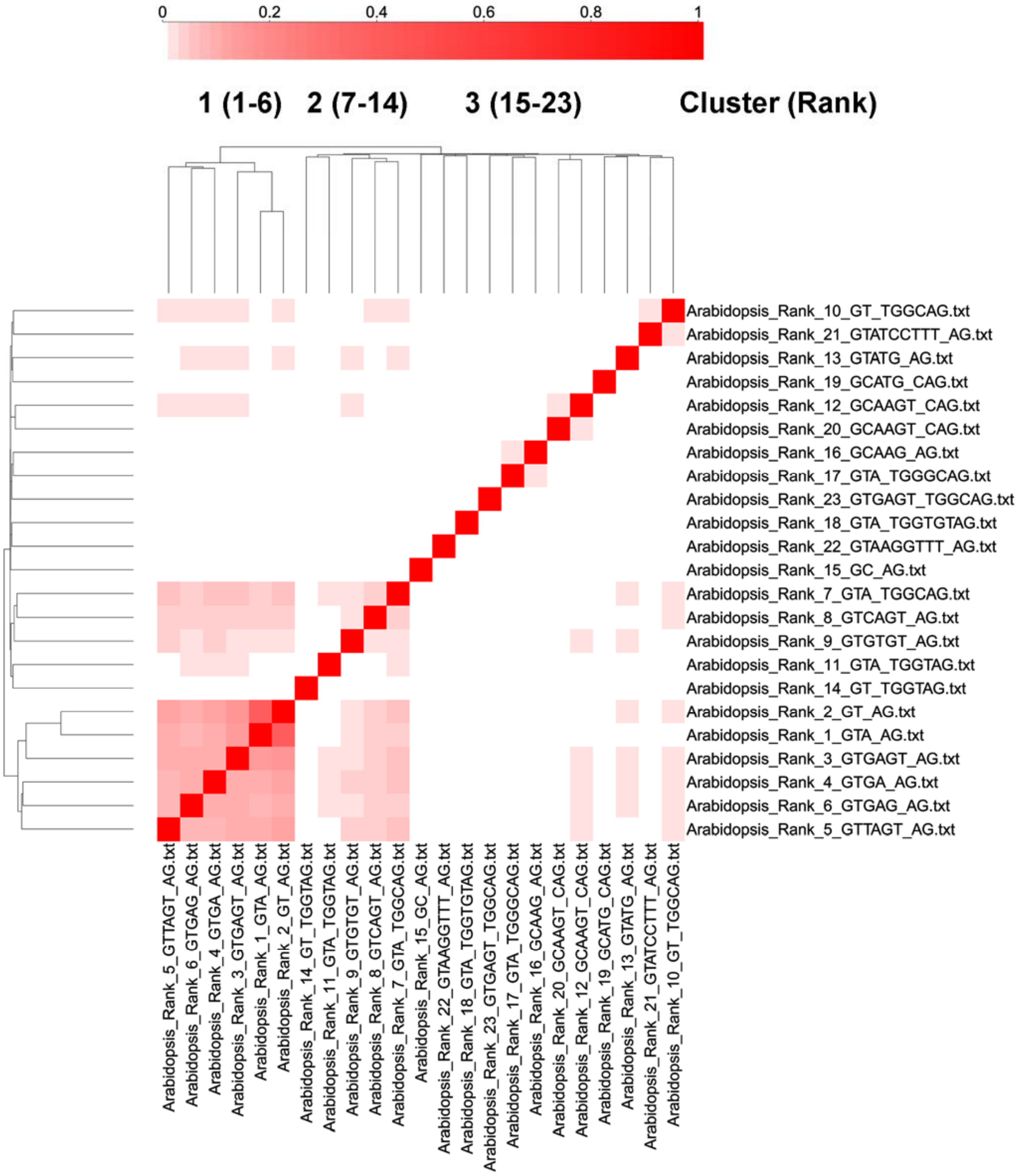

Supplementary Figure 5

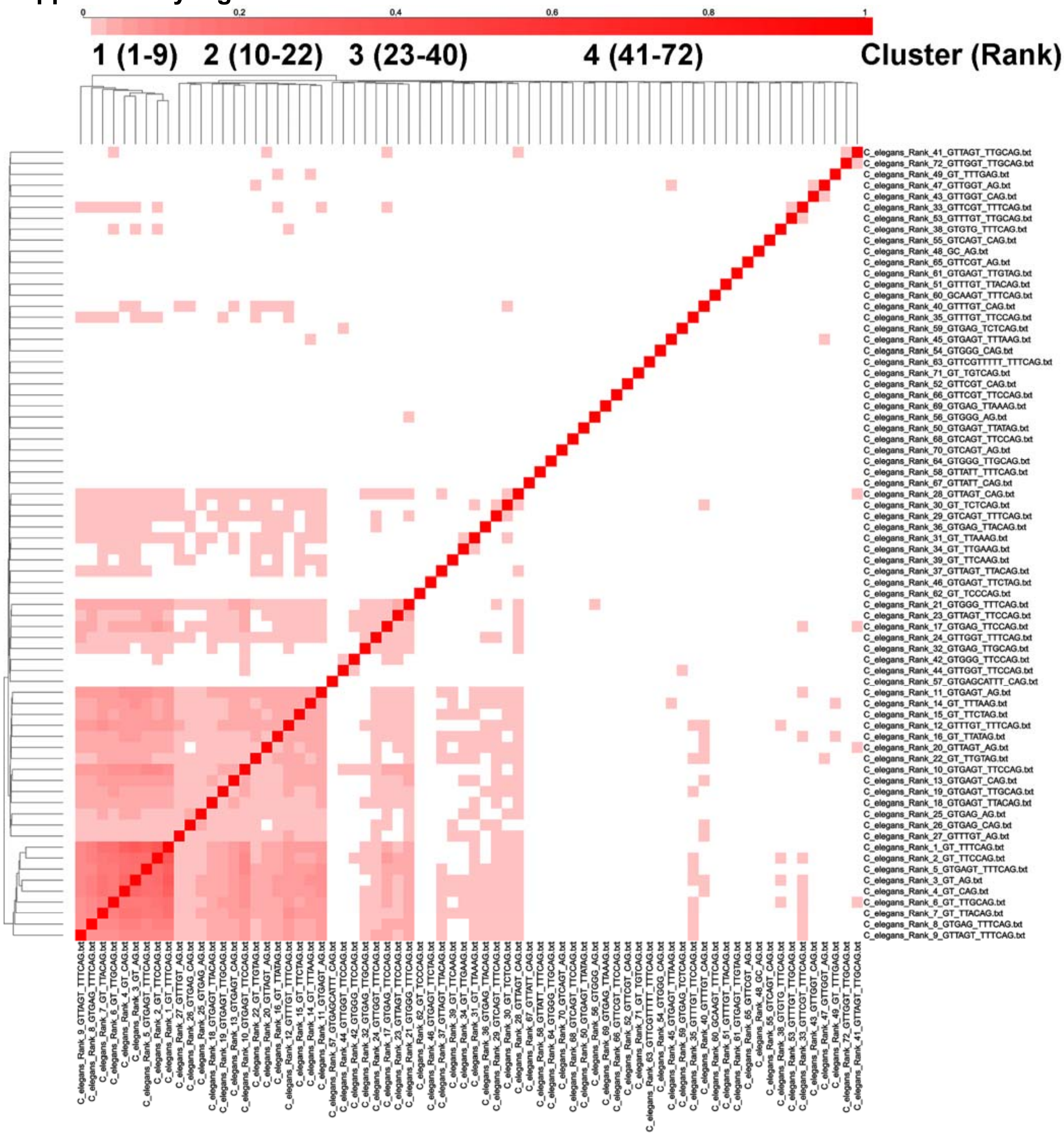

Supplementary Figure 6

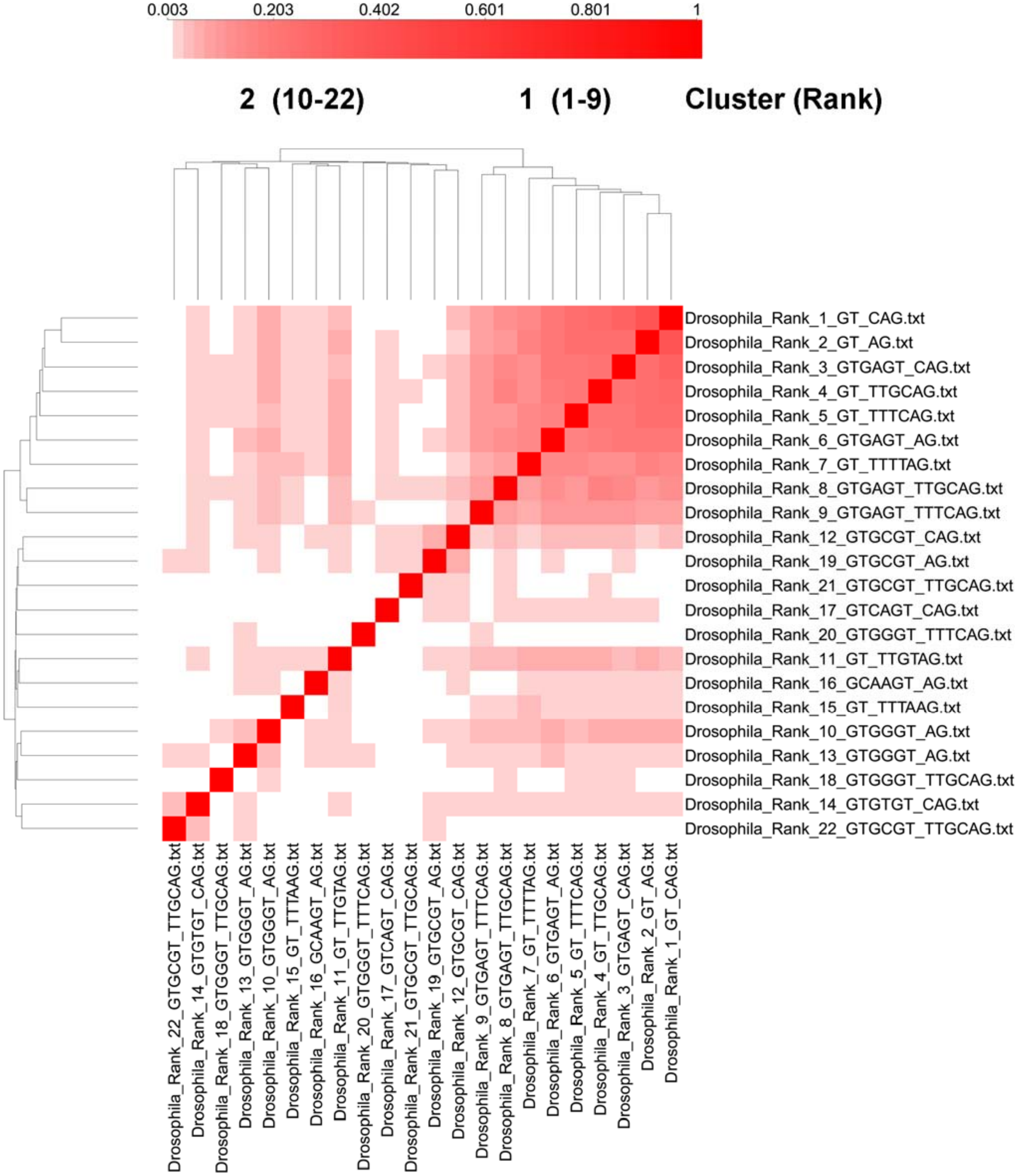

Supplementary Figure 7

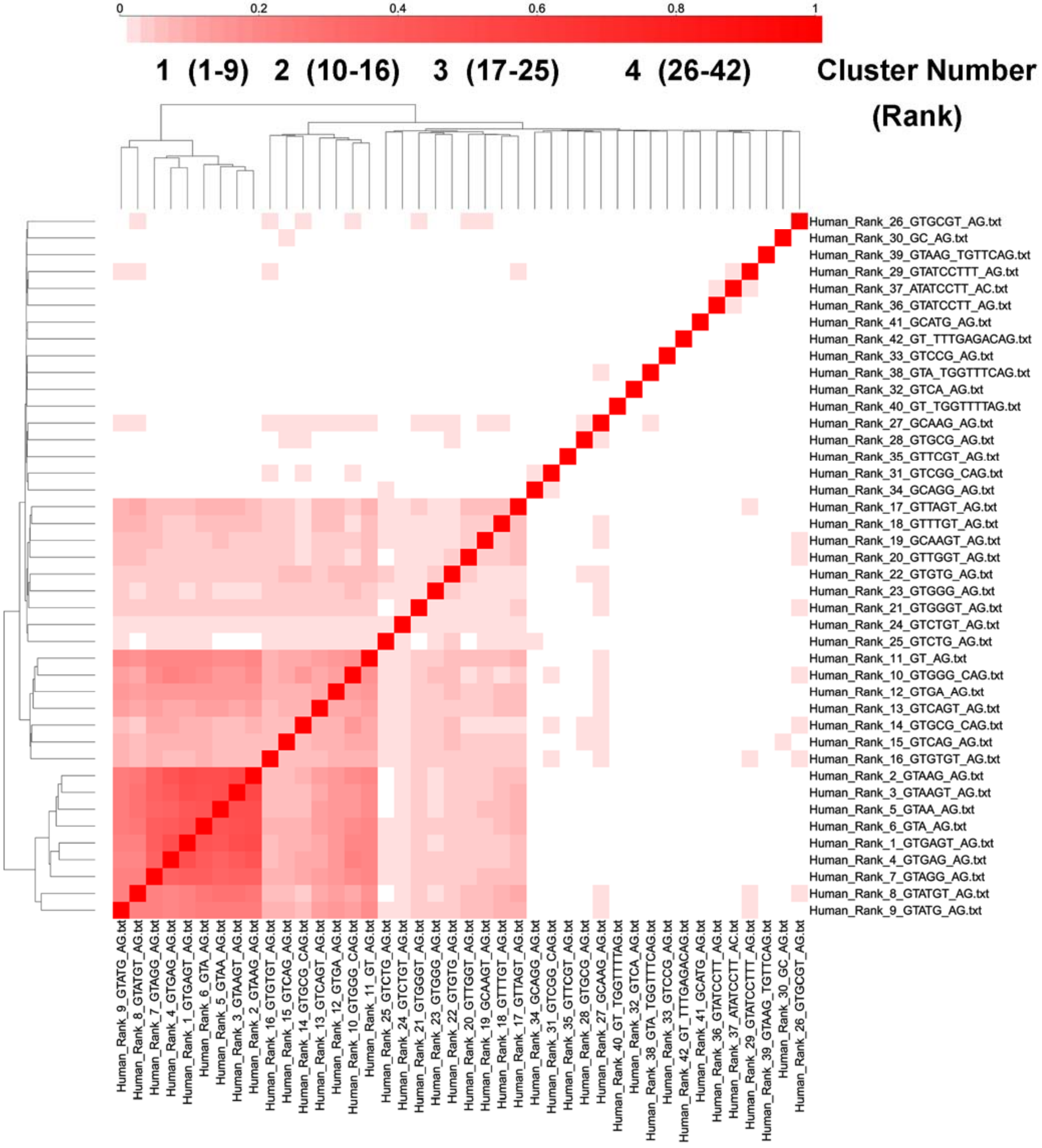

Supplementary Figure 8

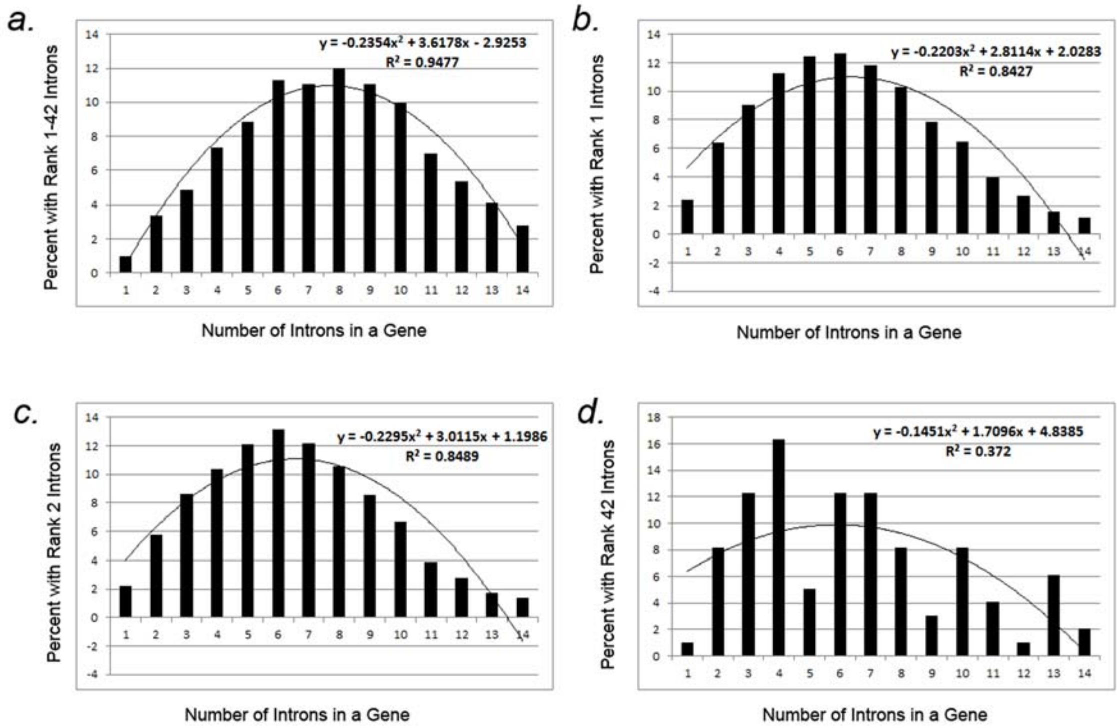

Supplement: Supplementary file 1 [file DataSheet1.pdf]
