# Supplementary material for: A Bioinformatics-Based Alternative mRNA Splicing Code that May Explain Some Disease Mutations Is Conserved in Animals
Source: Front Genet. 2017 Apr 11;8:38. doi: 10.3389/fgene.2017.00038 (PMC5387049; doi:10.3389/fgene.2017.00038)
Supplement: Supplementary file 2 [file Table1.DOC]

**Supplement Tables**

**Supplement Table 1**. *Intron types annotated in the five most well-studied genomes.* Alternative splicing classes occurrence numbers in H. sapiens (human), M. musculus (mouse), D. melanogaster (Drosophila), C. elegans and A. thaliana are shown.

| **36 Intron Types**  **(Abbreviation)** | **Count** | | | | |
| --- | --- | --- | --- | --- | --- |
|  | **H. sapiens** | **M.musculus** | **D. melanogaster** | **C. Elegans** | **A. Thaliana** |
| Total | 215155 | 200270 | 56509 | 111780 | 127530 |
| 1. A3-A3 | 358 | 7555 | 686 | 937 | 1182 |
| 1. A3-A5 | 136 | 8905 | 370 | 507 | 331 |
| 1. A3-APA | 73 | 346 | 0 | 0 | 112 |
| 1. A3-ME | 15 | 0 | 0 | 0 | 3 |
| 1. A3-R | 2428 | 3049 | 2467 | 4139 | 1487 |
| 1. A3-S | 551 | 10579 | 457 | 655 | 170 |
| 1. A5-A3 | 1313 | 4968 | 2169 | 693 | 961 |
| 1. A5-A5 | 211 | 4472 | 332 | 383 | 273 |
| 1. A5-APA | 349 | 1363 | 338 | 674 | 597 |
| 1. A5-ME | 9 | 0 | 0 | 0 | 5 |
| 1. A5-R | 2191 | 382 | 1476 | 461 | 575 |
| 1. A5-S | 1125 | 9024 | 829 | 588 | 188 |
| 1. APr-A3 | 1145 | 2309 | 1680 | 1335 | 978 |
| 1. APr-A5 | 171 | 1043 | 179 | 0 | 97 |
| 1. APr-APA | 63 | 0 | 0 | 0 | 115 |
| 1. APr-ME | 9 | 0 | 0 | 0 | 1 |
| 1. APr-R | 3741 | 1518 | 2030 | 710 | 599 |
| 1. APr-S | 2155 | 2429 | 1104 | 928 | 115 |
| 1. ME-A3 | 14 | 0 | 0 | 0 | 5 |
| 1. ME-A5 | 8 | 0 | 0 | 0 | 1 |
| 1. ME-APA | 2 | 0 | 0 | 0 | 1 |
| 1. ME-ME | 14 | 0 | 0 | 0 | 2 |
| 1. ME-R | 177 | 0 | 100 | 0 | 12 |
| 1. ME-S | 14 | 0 | 0 | 0 | 0 |
| 1. R-A3 | 3239 | 596 | 1782 | 777 | 1270 |
| 1. R-A5 | 1496 | 3241 | 2038 | 1564 | 1015 |
| 1. R-APA | 772 | 377 | 243 | 331 | 232 |
| 1. R-ME | 168 | 0 | 108 | 27 | 13 |
| 1. R-R | 168938 | 69770 | 31463 | 89272 | 115948 |
| 1. R-S | 4782 | 1185 | 1087 | 551 | 263 |
| 1. S-A3 | 1019 | 8211 | 706 | 999 | 185 |
| 1. S-A5 | 313 | 10719 | 350 | 373 | 71 |
| 1. S-APA | 1310 | 2207 | 350 | 516 | 151 |
| 1. S-ME | 9 | 0 | 0 | 0 | 0 |
| 1. S-R | 5345 | 1404 | 1560 | 699 | 309 |
| 1. S-S | 11492 | 44304 | 2483 | 4447 | 263 |

**Supplement Table 2.** *The number of intron consensus sequences in animals and plants from smallest to largest.*

**Common Name,** the version of the genome that was analyzed by SnpEff [^25^](#_ENREF_25). **Number Introns,** the total number of introns in the organism. **Consensus Sequences,** the number of paired consensus sequences that are present in at least 100 introns. **Most Common,** the most common paired consensus sequence in the organism. **Best U12-like,** the U12-like consensus sequence that is a best match to the canonical U12 sequence in humans. Note that a consensus sequence must be present in at least 100 introns to be counted. **None**, the U12 –like sequence was not present in at least 100 introns (many U12-like introns are not included in this study because they are present in fewer than 100 introns).

| **Common Name** | **Genus Species** | **Genome Version** | **Number Introns** | **Consensus Sequences** | **Most Common** | **Best U12-like** |
| --- | --- | --- | --- | --- | --- | --- |
| baker's yeast | Saccharomyces cerevisiae | EF4.71 | 282 | 1 | guaugu_ag | none |
| zebra finch | Taeniopygia_guttata | taeGut3.2.4.71 | 54,176 | 11 | guaa_ag | guauccuu_cag |
| sea lamprey | Petromyzon marinus | Pmarinus_7.0.71 | 24,727 | 14 | gu_cag | none |
| common shrew | Sorex araneus | COMMON_SHREW1.71 | 52,620 | 19 | gua_ag | guauccuu_cag |
| Philippine tarsier | Tarsius syrichta | tarSyr1.71 | 62,650 | 20 | gua_ag | guauccuu_cag |
| wild turkey | Meleagris gallopavo | UMD2.71 | 56,168 | 21 | gua_ag | none |
| thale cress | Arabidopsis thaliana | athalianaTair10 | 127,530 | 23 | gu_ag | guauccuuu_ag |
| solitary sea squirt | Ciona savignyi | CSAV2.0.71 | 61,597 | 28 | gu_cag | none |
| fruit fly | Drosophila melanogaster | BDGP5.71 | 56,509 | 29 | gu_ag | gucagu_ag |
| vase tunicate | Ciona intestinalis | KH.71 | 75,187 | 33 | guaa_ag | none |
| horse | Equus caballus | EquCab2.71 | 114,781 | 34 | guaag_ag | guauccuuu_ag |
| mouse lemur | Microcebus murinus | micMur1.71 | 81,422 | 34 | guaag_ag | guauccuu_cag |
| tammar wallaby | Macropus eugenii | Meug_1.0.71 | 63,787 | 35 | guaag_ag | guauccuu_cag |
| European honey bee | Apis mellifera | amel2_cuff | 77,778 | 37 | gua_ag | guauccuuu_ag |
| chicken | Gallus gallus | Galgal4.71 | 130,831 | 38 | guaag_ag | guauccuu_cag |
| little brown bat | Myotis lucifugus | Myoluc2.0.71 | 94,423 | 39 | guaa_ag | guauccuuu_ag |
| Chinese softshell turtle | Pelodiscus sinensis | PelSin | 113,151 | 40 | guaag_ag | guauccuuu_ag |
| Gray short-tailed opposum | Monodelphis domestica | BROAD05.71 | 146,772 | 40 | guaagu_ag | guauccuuu_ag |
| medaka | Oryzias latipes | MEDAKA1.71 | 107,118 | 41 | gugag_cag | guauccuu_cag |
| domestic mouse | Mus musculus | GRCm38.71 | 200,270 | 41 | guaag_ag | guauccuuu_ag |
| human | Homo sapiens | hg19 | 215,155 | 42 | gugagu_ag | guauccuuu_ag |
| thirteen-lined ground squirrel | Ictidomys tridecemlineatus | spetri2.71 | 117,289 | 43 | guaag_ag | guauccuuu_ag |
| freshwater pufferfish | Tetraodon nigroviridis | TETRAODON8.71 | 97,364 | 43 | gu_cag | guauccuu_cag |
| Giant panda | Ailuropoda melanoleuca | ailMel1.71 | 98,357 | 43 | guaag_ag | guauccuuu_ag |
| American chameleon | Anolis carolinensis | AnoCar2.0.71 | 127,794 | 44 | guaag_ag | guauccuuu_ag |
| small-eared galago | Otolemur garnettii | OtoGar3.71 | 120,280 | 45 | guaag_ag | guauccuuu_ag |
| Norwegian rat | Rattus norvegicus | Rnor_5.0.71 | 162,093 | 45 | guaag_ag | guauccuuu_ag |
| Macaque | Macaca mulatta | MMUL_1.71 | 158,008 | 45 | guaag_ag | guauccuuu_ag |
| Sumatran orangutan | Pongo abelii | PPYG2.71 | 150,216 | 46 | guaag_ag | guauccuuu_ag |
| Nile tilapia | Oreochromis niloticus | Orenil1.0.71 | 176,612 | 46 | guaa_ag | guauccuu_cag |
| domestic cat | Felis catus | Felus_catus_6.2.71 | 115,833 | 46 | guaa_ag | guauccuuu_ag |
| white-cheeked gibbon | Nomascus leucogenys | Nleu1.0.71 | 114,903 | 46 | guaagu_ag | guauccuuu_ag |
| chimpanzee | Pan troglodytes | CHIMP2.1.4.71 | 153,635 | 46 | guaagu_ag | guauccuuu_ag |
| common bottlenose dolphin | Tursiops truncatus | turTru1.71 | 114,568 | 46 | guaa_ag | guauccuuu_ag |
| Tasmanian devil | Sarcophilus harrisii | DEVIL7.0.71 | 108,455 | 46 | gugag_ag | guauccuuu_ag |
| Domestic dog | Canis familiaris | CanFam3.1.71 | 161,608 | 48 | guaagu_ag | guauccuuu_ag |
| cow | Bos taurus | UMD3.1.71 | 145,418 | 49 | gugag_ag | guauccuuu_ag |
| Guinea pig | Cavia porcellus | cavPor3.71 | 102,700 | 50 | guaag_ag | guauccuuu_ag |
| Zebra fish | Danio rerio | Zv9.71 | 202,834 | 51 | guaa_ag | guauccuu_ag |
| southern platyfish | Xiphophorus maculatus | Xipmac4.4.2.71 | 154,330 | 51 | gu_ag | guauccuu_cag |
| European rabbit | Oryctolagus cuniculus | oryCun2.71 | 109,457 | 51 | guaag_ag | guauccuuu_ag |
| Western clawed frog | Xenopus tropicalis | JGI_4.2.71 | 108,674 | 51 | guaag_ag | guauccuuu_ag |
| domestic ferret | Mustela puorius | MusPutFur1.0.71 | 154,773 | 52 | gugag_cag | guauccuuu_ag |
| wild pig | Sus scrofa | Sscrofa10.2.71 | 121,311 | 54 | gugag_cag | guauccuuu_ag |
| African elephant | Loxodonta africana | loxAfr3.71 | 114,565 | 59 | gugag_ag | guauccuuu_ag |
| Coelocanth | Latimeria chalumnae | LatCha1.71 | 107,544 | 61 | guaag_ag | guauccuuu_ag |
| mountain gorilla | Gorilla gorilla | gorGor3.1.71 | 151,559 | 63 | guaag_ag | guauccuuu_ag |
| round worm | Caenorhabidtis elegans | WBce1235.71 | 111,780 | 69 | gu_uuucag | guucguuuuu_uuucag |
| pufferfish | Takifugu rubripes | FUGU4.71 | 150,616 | 71 | gugag_cag | guauccuu_cag |
| marmoset | Callithrix jacchus | C_jacchus3.2.1.71 | 184,882 | 95 | gugagu_ag | guauccuu_ag |

**Supplementary Table 3.** (Excel format online) Interpretation of 96 mutations that alter alternative mRNA splicing in humans using the alternative RNA splicing code.

**
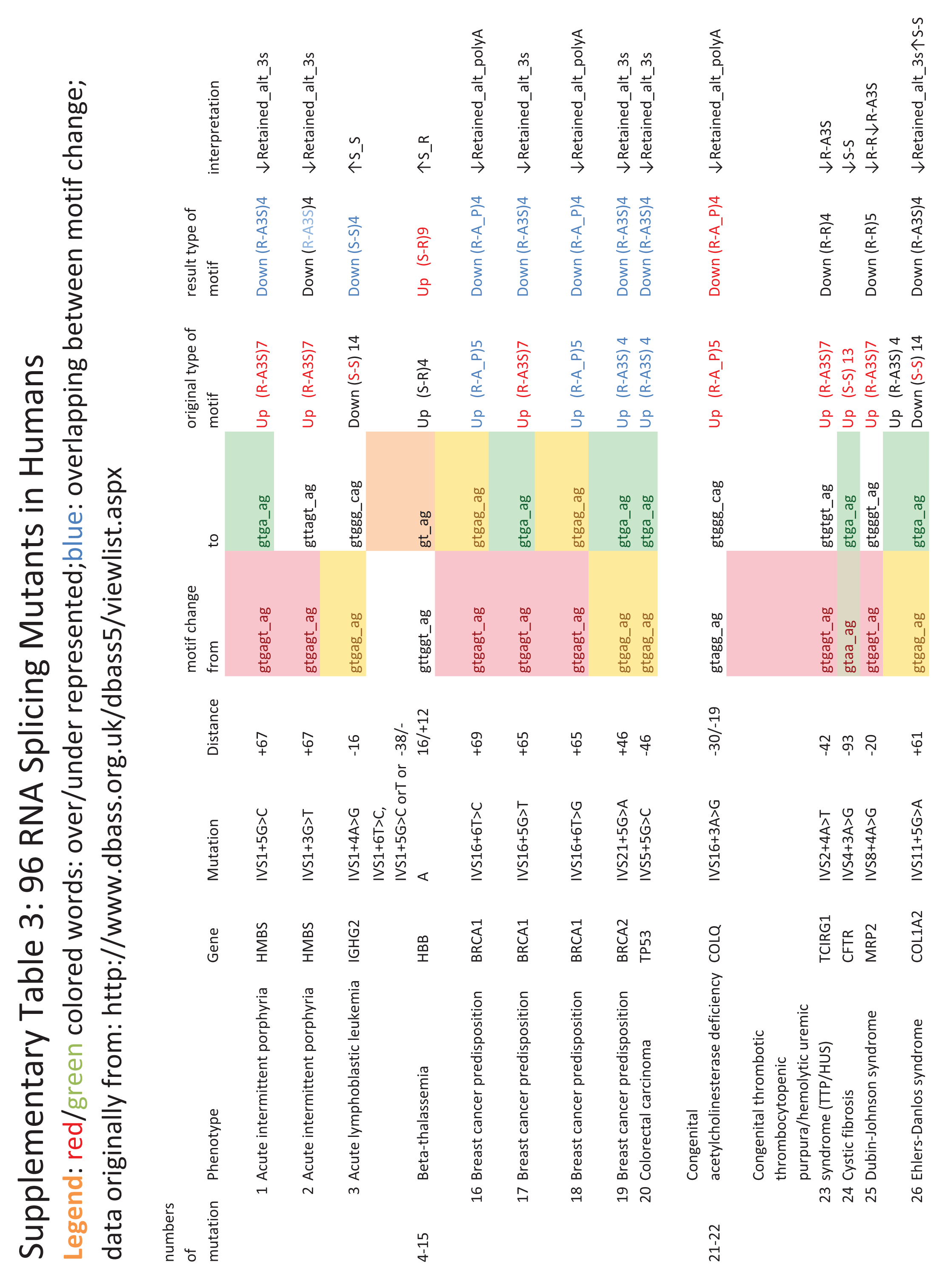

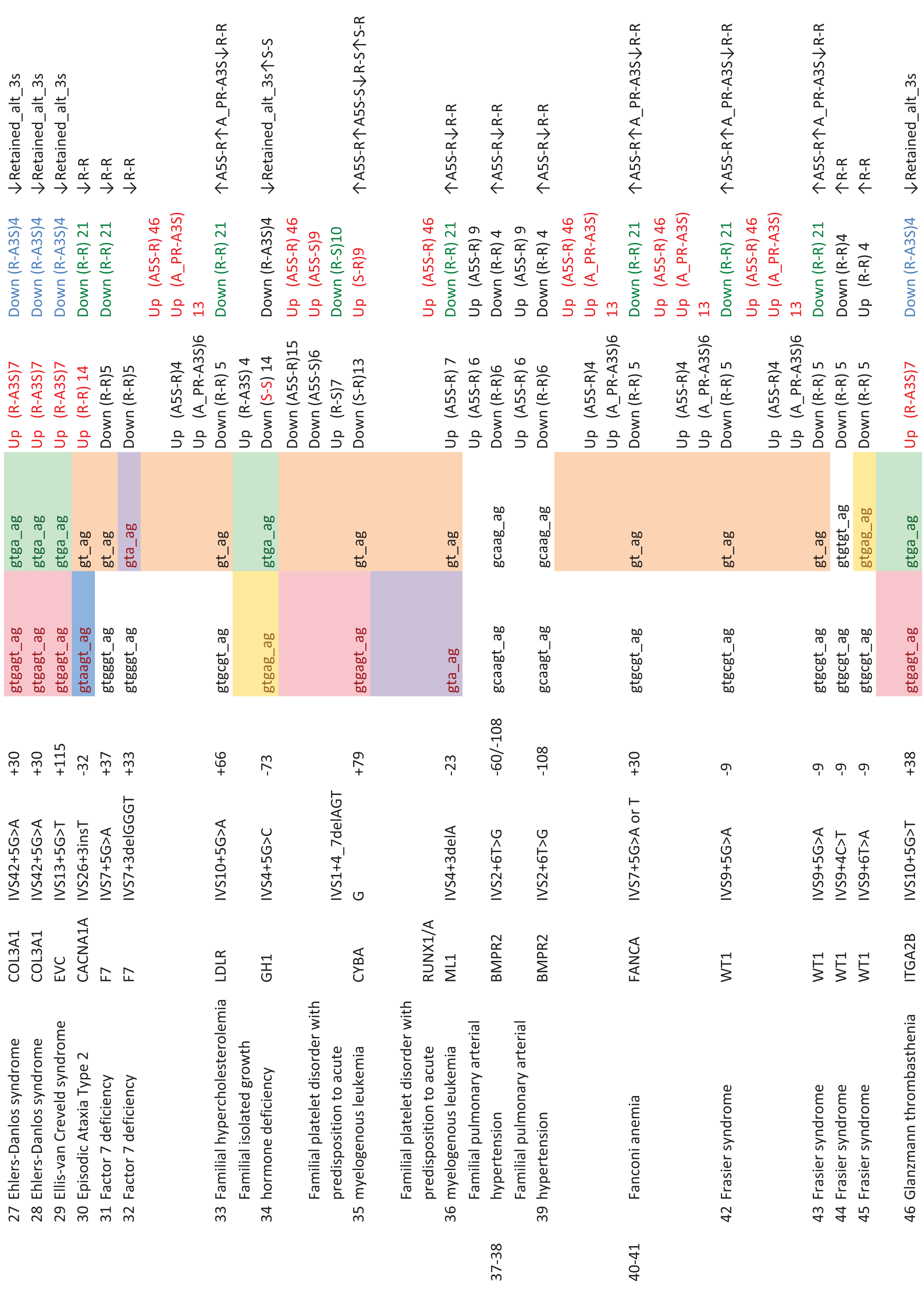

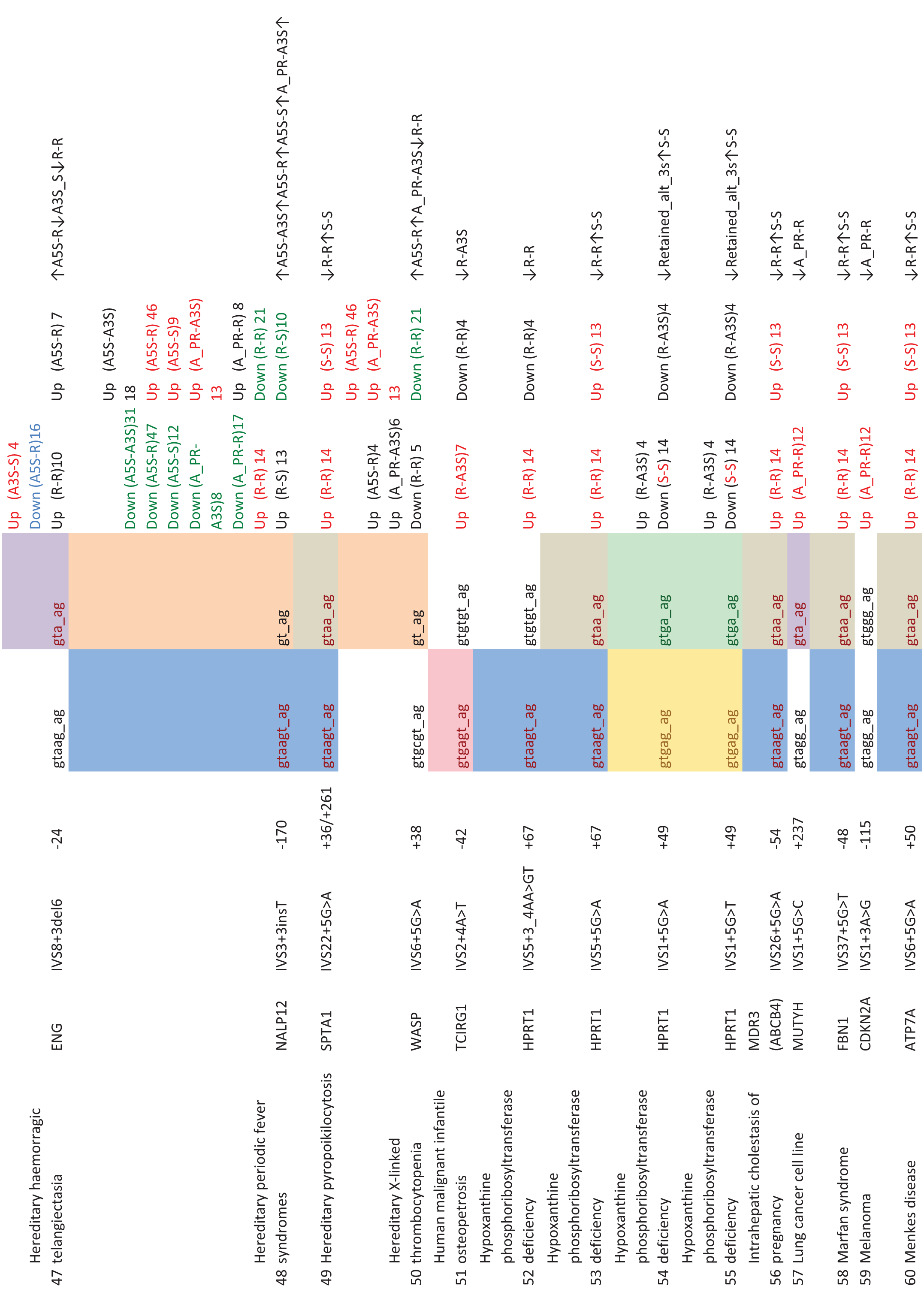

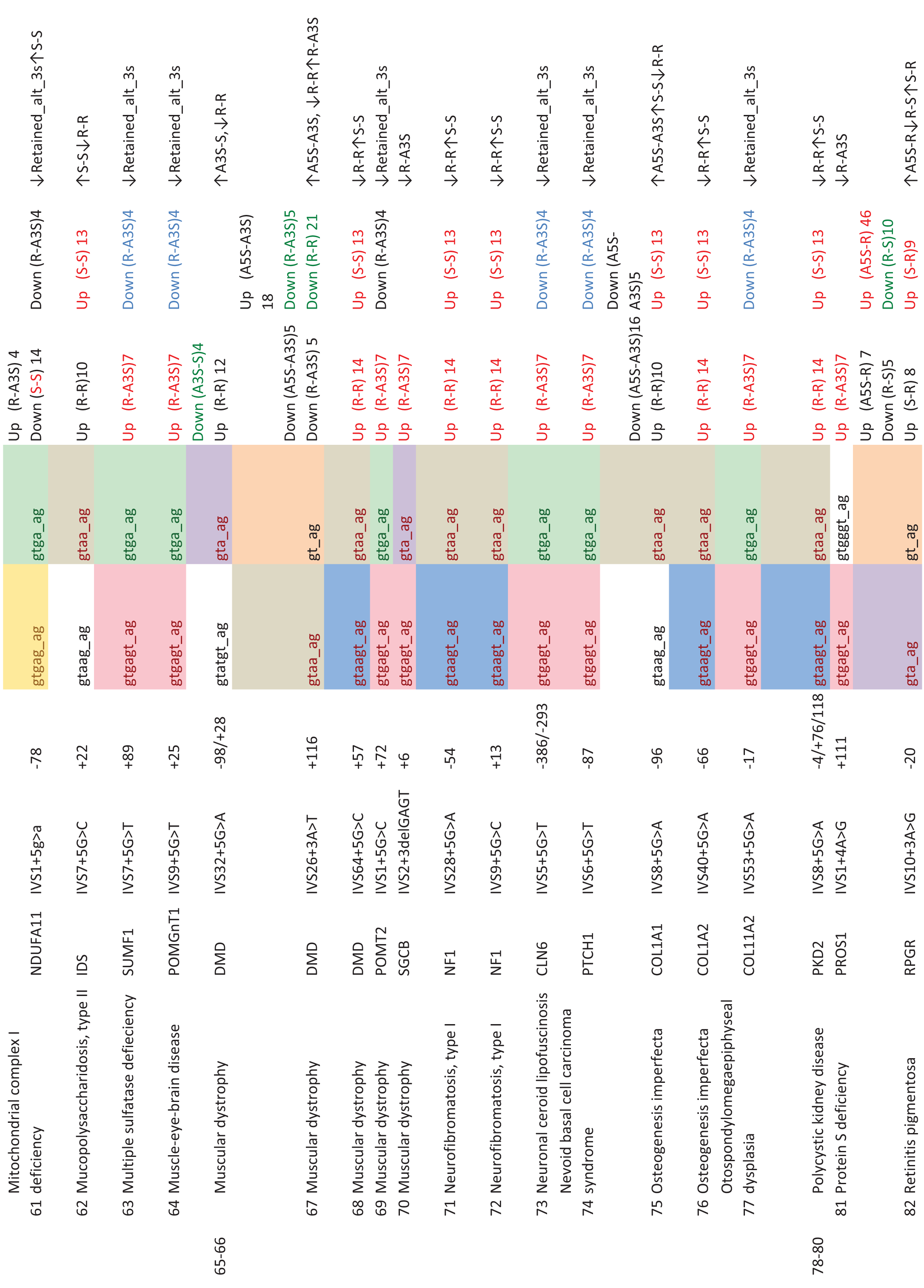

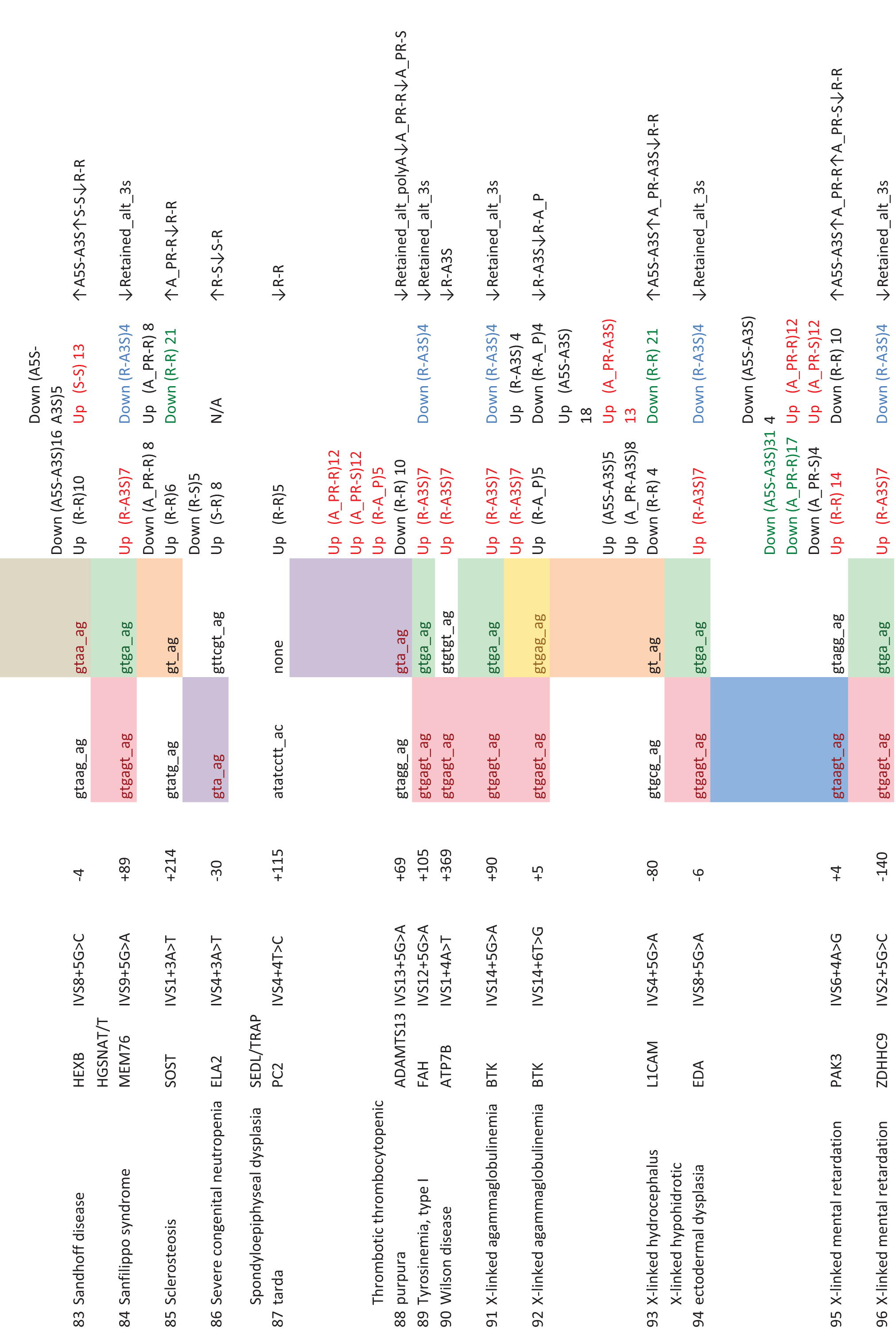
**

**Supplementary Table S4.A.** Parameters in common in all HTGM analyses

| **Parameter** | **Value** |
| --- | --- |
| **Root category for biological process** | GO:0008150 biological process |
| **GO database version** | January 2011 |
| **GoMiner version** | web HTGM |
| **GoMiner build** | 291 |
| **Evidence code level** | 1 |
| **Cross reference** | false |
| **Synonym** | false |
| **Randomizations** | 100 |
| **Smallest category size** | 5 |
| **Largest category size** | 500 |
| **FDR threshold** | 0.01 |
| **p-value threshold** | 0.01 |

**Supplementary Table S4.B.** Parameters that vary across different HTGM analyses

| **Parameter** | **human** | **mouse** | **D. melanogaster** | **C. elegans** | **A. thaliania** |
| --- | --- | --- | --- | --- | --- |
| **Datasource** | UniProtKB | MGI | FB | WB | TAIR |
| **Organism** | 9606 | 10090 | 7227 | 6239 | 3703 |
